# Gas-permeable and stretchable nanoscale anisotropic conductive film for room-temperature, pressure-free interconnections

**DOI:** 10.1126/sciadv.aeh6203

**Published:** 2026-09-18

**Authors:** Shumpei Katayama, Lulu Sun, Tomoki Shigehara, Daishi Inoue, Daisuke Hashizume, Shinjiro Umezu, Kenjiro Fukuda, Sunghoon Lee, Takao Someya

**Affiliations:** ^1^ RIKEN Center for Emergent Matter Science (CEMS), 2-1 Hirosawa, Wako, Saitama 351-0198, Japan.; ^2^ Department of Modern Mechanical Engineering, Waseda University, 3-4-1 Okubo, Shinjuku-ku, Tokyo 169-8555, Japan.; ^3^ Thin-Film Device Laboratory, RIKEN, 2-1 Hirosawa, Wako, Saitama 351-0198, Japan.; ^4^ Division of Electrical, Electronic and Infocommunications Engineering, Graduate School of Engineering, The University of Osaka, 2-1 Yamada-Oka, Suita, Osaka 565-0871, Japan.; ^5^ Department of Electrical Engineering and Information Systems, The University of Tokyo, 7-3-1 Hongo, Bunkyo-ku, Tokyo 113-8656, Japan.

## Abstract

As stretchable electronics advance in functionality and application scope, interconnection technologies have become critical. Devices in wearable and soft-robotic systems require thinness, stretchability, gas permeability, and damage-free bonding conditions. Accordingly, interconnection technologies must exhibit similar properties without compromising device performance. Conventional interconnection approaches rarely satisfy these requirements simultaneously owing to constraints such as typical thickness of several micrometers, low gas permeability, and the requirement for harsh bonding conditions (heat, pressure, or ultraviolet). This study presents an ultrathin, highly stretchable, gas-permeable nanoscale anisotropic conductive film (ACF) enabling damage-free bonding. The proposed ACF consists of patterned disks of silver nanowires infiltrated with styrene-ethylene-butylene-styrene block copolymer. The resulting nanoscale film (∼300-nanometer thickness) exhibits mechanical and electrical stretchability exceeding 500% strain with a water vapor transmission rate of 1200 grams per square meter per day. A bonding process based on liquid volatilization enables conformal adhesion to uneven surfaces without heat or external pressure, allowing device-to-skin and device-to-device interconnections. Light-emitting diodes integrated with stretchable wiring (500-micrometer pitch) using the proposed ACF maintained electrical conduction and interwire insulation even under 500% strain.

## INTRODUCTION

The increasing functionality of individual stretchable electronic devices has expanded their application scenarios, making the performance of interconnection technologies critical. Stretchable electronics are required in diverse and expanding fields, including wearable devices for continuous biomedical monitoring in health care (*1*–*4*), motion analysis (*5*, *6*), and input (*7*–*9*) and display technologies (*10*–*12*). These requirements also extend to soft robotics with applications such as nondamaging gripping (*13*–*15*), replication of biological functions (*16*, *17*), and medical robots that directly contact the human body (*18*–*21*). Accordingly, the functional requirements arising from these expanding application fields include low stiffness for intimate contact, extreme thinness (a few micrometers or less), high stretchability exceeding several hundred percent, the use of biocompatible materials, and gas permeability suitable for long-term on-body operation. To satisfy these diverse requirements and achieve system-level functional integration, interconnection technologies enabling bonding between devices fabricated on different film substrates are essential (*22*, *23*). Specifically, the key requirements for such interconnection technologies include thinness, low stiffness, high electrical stretchability, biocompatibility, a high water vapor transmission rate (WVTR), and mild bonding conditions to prevent damage to the bonding substrate or mounted devices.

Various methods for conductive bonding on flexible and stretchable substrates have been proposed. These methods generally fall into two categories: direct bonding and bonding using anisotropic conductive films (ACFs). One direct bonding approach involves embedding metal particles in an adhesive polymer matrix to achieve adhesion at the electrode interface and is categorized as direct bonding because the conductive path is established directly at the interface (*24*). For example, Au nanoparticles integrated into elastomeric substrates provide robust adhesion at the substrate interface, facilitating continuous monitoring of physiological signals. Another method involves direct bonding of metal thin films through plasma treatment (*23*, *25*). Au or Ag layers deposited on thin polymeric substrates, such as parylene, have been successfully bonded to integrate complex components such as organic light-emitting diodes (LEDs) and organic photodetectors. These direct bonding approaches are defined by connections through direct contact at the electrode interface and typically require precise material compatibility between the bonded surfaces. Moreover, the resulting conductive path is isotropic within the bonding plane. By contrast, ACFs serve as intermediate conductive adhesive layers for electrodes, enabling their application to diverse substrates. Conventional ACF tapes have been widely adopted as an industrial standard for electronic packaging. They are typically fabricated by randomly dispersing conductive particles within an epoxy resin. Thermal pressing produces in-plane insulation and through-thickness conductivity (*26*–*29*). A stretchable ACF has been proposed to extend its application range. Specific examples include ACFs that achieve high stretchability by incorporating nanofluids into epoxy resins (*30*); stretchable ACFs fabricated by periodically embedding metal particles in thermoplastic block-copolymer films (*31*); and ACFs that achieve stable electrical contact during stretching and low contact resistance by periodically incorporating liquid metal into epoxy resins (*32*).

However, conventional stretchable ACFs cannot simultaneously achieve the thinness, high stretchability, gas permeability, and damage-free bonding conditions required for next-generation stretchable electronics. Conventional ACFs are typically several micrometers thick. This thickness compromises the flexibility and low stiffness required for submicrometer-scale stretchable electronics. Furthermore, conventional ACFs are typically bonded by curing the resin under pressure, heat, or ultraviolet (UV) irradiation (*33*). Such processes can cause physical damage or UV-induced degradation of the bonding substrates. Moreover, these processes fundamentally limit direct integration onto uneven, soft, or stimulation-sensitive surfaces, often requiring additional preprocessing before mounting. In addition to their typical thickness, conventional ACFs often use low-WVTR materials, which can compromise the high gas permeability required for stretchable electronics. The simultaneous satisfaction of all these requirements remains an unresolved challenge in current ACF technologies.

In this study, we present an ultrathin stretchable ACF that simultaneously achieves extreme thinness (∼300 nm), high stretchability (mechanical and electrical stretchability >500%), high gas permeability (WVTR of 1200 g/m^2^ per day), and room-temperature, pressure-free damage-free bonding for next-generation stretchable electronics. The ultrathin stretchable ACF was fabricated by periodically arranging silver nanowire (AgNW) disks via spray coating and infiltrating the interior with a styrene-ethylene-butylene-styrene (SEBS) block copolymer. The AgNW disks deform seamlessly during stretching, achieving mechanical and electrical stretchability exceeding 500% while maintaining a high WVTR of 1200 g/m^2^ per day at ∼300-nm thickness. Room-temperature pressure-free bonding is achieved using a wet-volatile process (*34*), a damage-free bonding method that uses liquid volatilization to enable conformal adhesion. The ultrathin stretchable ACF maintains flexibility even after bonding 2-μm-thick film electrodes and conforms tightly to surfaces with microscale roughness. We also demonstrated high electrical stability under stretching when the LEDs were bonded to stretchable electronics. These characteristics enable direct application to the skin and device-to-device interconnections.

## RESULTS

### Structure of the ultrathin stretchable ACF

**Fig. 1.**
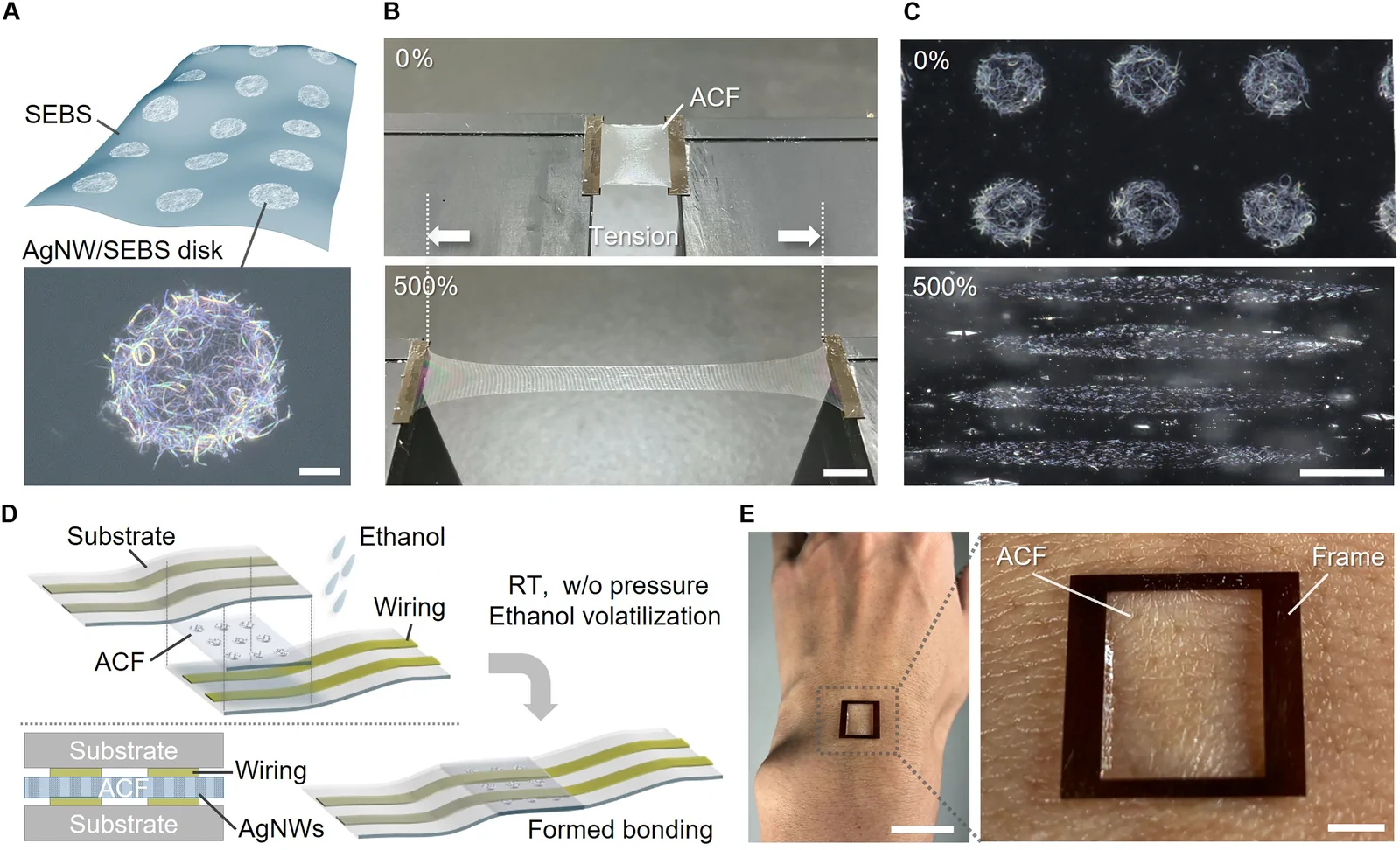
Structure and bonding method of the ultrathin stretchable ACF. (**A**) Schematic illustration of the ultrathin stretchable ACF structure and optical image of the AgNW disks. Scale bar, 10 μm. (**B**) Optical image of the ACF under tensile strain. Scale bar, 5 mm. Contrast has been enhanced for improved visibility. (**C**) Top-view optical images of the structure bonded onto a thick SEBS substrate under stretching at strains of 0 and 500%. Scale bar, 50 μm. (**D**) Schematic illustration of the bonding process of parylene-based Cr/Au films via the wet-volatile process using the ultrathin stretchable ACF. RT, room temperature. (**E**) Ultrathin stretchable ACF integrated onto skin via the wet-volatile process. Scale bars, 10 mm (left) and 3 mm (right).

The structure of the ultrathin stretchable ACF with AgNW/SEBS conductive regions is illustrated in Fig. 1A. The ultrathin stretchable ACF consists of AgNW conductive and insulating regions arranged in uniform intervals within the SEBS film. The conductive regions were circular structures formed by AgNWs, with SEBS infiltrating both the spaces between and within the AgNW disks. This design ensures that the AgNW exposure on the film surface forms a through-thickness conductive path. Cross-sectional scanning electron microscopy (SEM) images (fig. S1) confirmed that the AgNWs were embedded within ∼300-nm-thick SEBS. The ultrathin ACF exhibited high stretchability, accommodating strains exceeding 500% (Fig. 1B and movie S1). The AgNW/SEBS conductive regions exhibited a stretchable structure, in which the circular pattern deformed in response to the stretching of the ultrathin stretchable ACF (Fig. 1C). The structure remained stable even after 1000 cycles at 500% strain (fig. S2).

The bonding of electrodes using the ultrathin stretchable ACF is performed using a wet-volatile process, a damage-free bonding method that uses liquid volatilization. The ultrathin stretchable ACF was sandwiched between two 2-μm-thick parylene films with Cr/Au (3.5/100 nm) wiring. Bonding was achieved by introducing a volatile liquid (such as ethanol), which was allowed to volatilize at room temperature without applying external pressure (Fig. 1D). Cross-sectional SEM and energy-dispersive x-ray spectroscopy (EDX) of the bonded sample confirmed uniform bonding while maintaining the integrity of the ultrathin stretchable ACF structure (figs. S3 and S4). Using this wet-volatile process, the ultrathin stretchable ACF can be directly integrated onto biological surfaces such as the skin (Fig. 1E). After removing the frame and conforming the ultrathin stretchable ACF to the skin, it became nearly imperceptible from the surface (fig. S5).

### The ultrathin stretchable ACF fabrication process and optimization

**Fig. 2.**
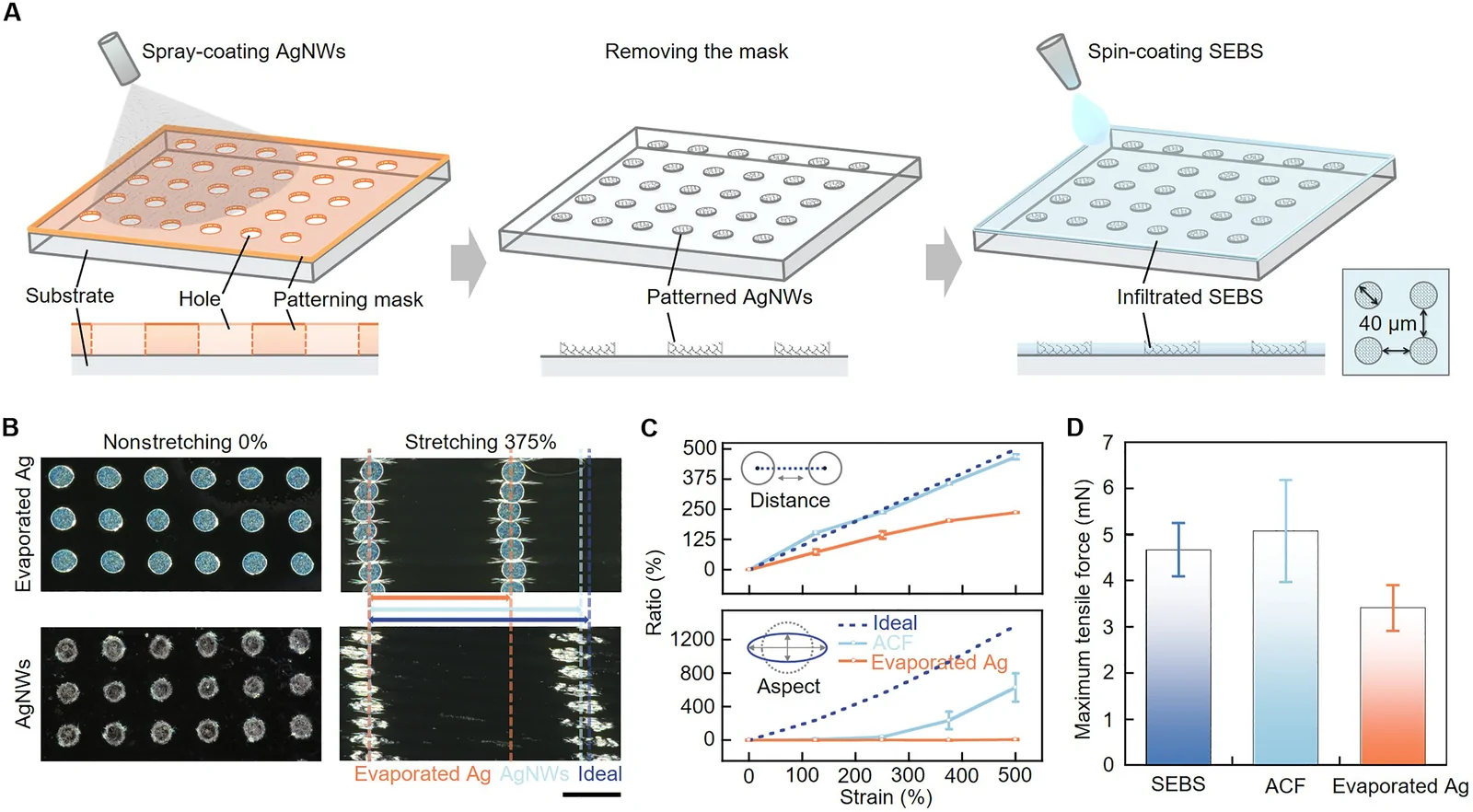
Ultrathin stretchable ACF fabrication process and optimization. (**A**) Schematic of the simplified ultrathin stretchable ACF fabrication process. The dimensions of the AgNW disk patterns are also provided. (**B**) Top-view optical images of samples using deposited silver and AgNW networks, shown before stretching (0%) and after stretching (375%). Dotted lines indicate the centers of the disks, and arrows indicate the center-to-center distances. Scale bar, 50 μm. (**C**) Center-to-center distance and the aspect ratio (horizontal width/vertical width) of the disks as a function of strain up to 500%, under the conditions specified in (B). Dotted lines represent the calculated values for ideal deformation. (**D**) Maximum tensile force sustained by each sample film before fracture. Bars represent the mean of three samples (*n* = 3), and error bars indicate the SD.

A simplified fabrication process for the ultrathin stretchable ACF is illustrated in Fig. 2A. First, the AgNW dispersion was spray coated onto a temporary substrate covered with a perforated patterning mask. Next, the mask was removed to leave AgNW disks on the substrate, which formed a conductive pattern. Last, an SEBS solution was drop cast and spin coated over this pattern to yield the final ACF structure: an array of circular AgNWs/SEBS composite regions, each 40 μm in diameter and separated by a 40-μm insulating gap.

We investigated the effect of the SEBS layer thickness, controlled by the SEBS concentration used for spin-coating, on the through-thickness electrical conductivity of the ACF. Low SEBS concentrations (3 to 12 wt %) resulted in low resistance (<20 Ω). By contrast, the resistance increased drastically (>10^6^ Ω) at an 18 wt % concentration (fig. S6). The surface SEM images (fig. S7) at a 45° tilt showed clear AgNW exposure on the disks of the 3 wt % sample, particularly near the edges. By contrast, the 18 wt % sample showed little to no exposure, with the AgNWs almost entirely covered by SEBS. Although the 12 wt % sample also exhibited AgNW exposure, the exposure was relatively limited compared to that of the 3 wt % sample. Cross-sectional SEM images confirmed that the AgNWs were distributed throughout the thickness of the SEBS layer. These results indicate that the through-thickness electrical conductivity of the ACF is strongly governed by the surface exposure of the AgNWs. Therefore, we conclude that SEBS concentrations of 12 wt % or less are suitable for ACF fabrication. The ACF fabricated using a 3 wt % SEBS solution was selected as the optimal condition because it provided maximum AgNW exposure and low resistance.

The mechanical performance of the ultrathin stretchable ACF was compared with that of a control sample fabricated with 500-nm-thick deposited silver instead of AgNWs. Top-view optical images of the ultrathin stretchable ACF and control samples at 0 and 375% applied strain are presented in Fig. 2B. Although the center-to-center distance of the disks in the ultrathin stretchable ACF achieved a strain of 359%, which is close to the ideal value for uniform film stretching, the control sample exhibited nonuniform and much lower strain of 203%. Over the 0 to 500% strain range, the disk-center-to-center distance in the ultrathin stretchable ACF increased proportionally with strain, whereas the control sample showed a clear deviation from the ideal linear relationship (Fig. 2C). In addition, the aspect ratio (horizontal width/vertical width) of the disks in the control sample showed negligible change. By contrast, the aspect ratio of the ultrathin stretchable ACF disks began to increase after 250% strain, suggesting that the AgNW disks deformed above a certain stress threshold, and subsequently reached 629% at 500% strain (Fig. 2C). The maximum tensile force before complete fracture was measured for each sample (Fig. 2D). The maximum tensile forces for the SEBS-only sample (without AgNWs) (4.67 ± 0.47 mN) and the ultrathin stretchable ACF (5.08 ± 0.90 mN) were comparable. Conversely, the control sample exhibited a lower maximum tensile force (3.41 ± 0.40 mN). This is likely because the nondeformed deposited silver disks create a distinct stress concentration at the interface with the SEBS regions, leading to the formation of holes. Furthermore, optical images confirmed a uniform color distribution in the stretched ultrathin ACF; however, numerous holes were observed in the control sample (fig. S8). These results confirm that the AgNW composite circular structure, which deforms together with the film, substantially improved the mechanical properties of the ultrathin stretchable ACF.

### The ultrathin stretchable ACF property evaluation

**Fig. 3.**
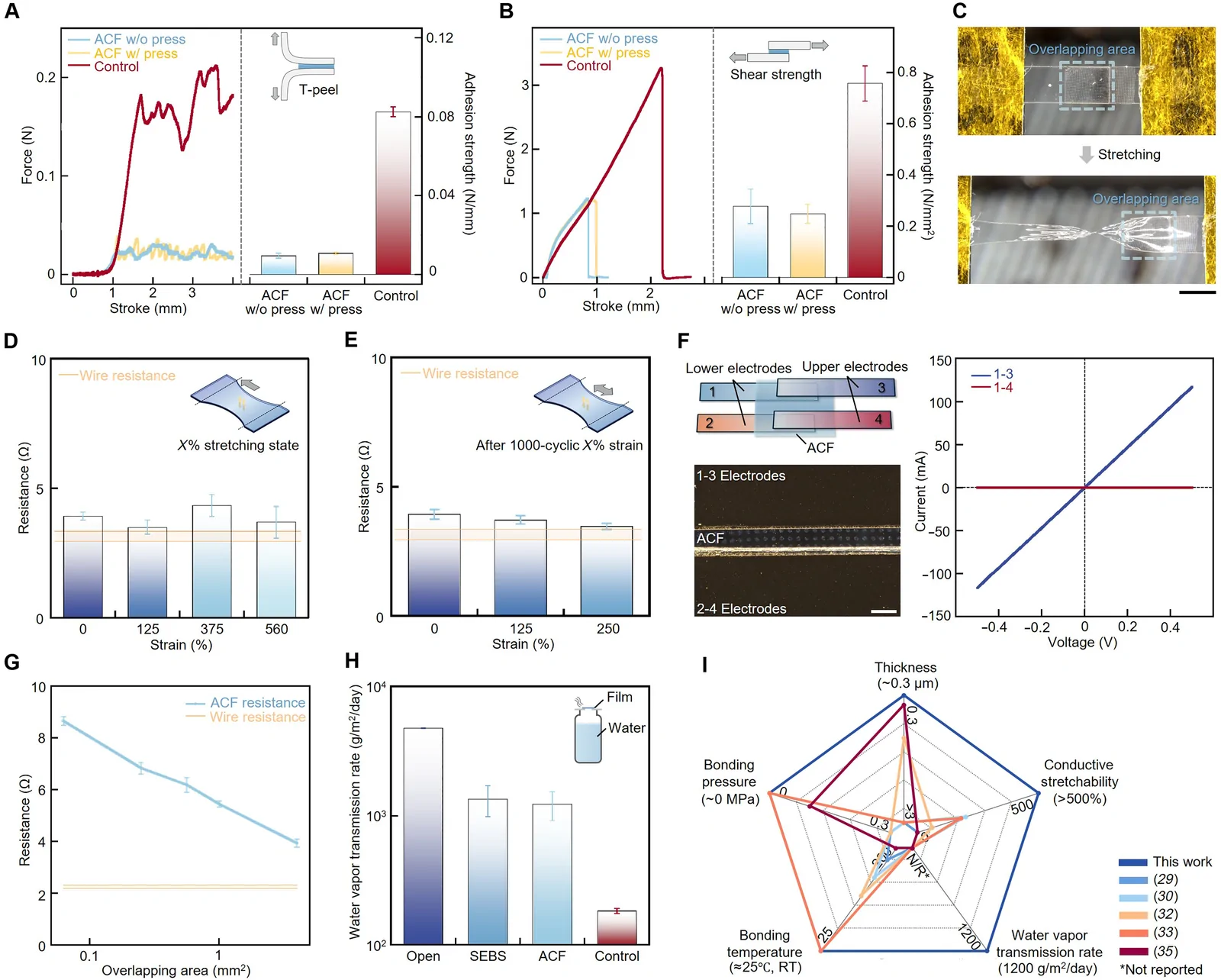
Characterization of the ultrathin stretchable ACF. (**A**) T-peel and (**B**) shear strength test results for the ultrathin stretchable ACF (bonded without/with pressure) compared with commercial ACF. (**C**) Photographs of 4-μm-thick parylene films bonded with the ultrathin stretchable ACF during tensile testing: (top) before stretching and (bottom) at film fracture. Scale bar, 2 mm. (**D**) Electrical resistance of bonded parylene-based Cr/Au films (bonding area of 2 mm by 2 mm) during stretching. (**E**) Electrical resistance of bonded parylene-based Cr/Au films (bonding area of 2 mm by 2 mm) in a relaxed state after 1000 stretching cycles. (**F**) Evaluation of insulation between electrodes with 200-μm spacing bonded with the ultrathin stretchable ACF. (Top left) Schematic of the bonding state, (bottom left) photograph of the bonding state, and (right) current during voltage application between electrodes. Scale bar, 250 μm. (**G**) Electrical resistance as a function of bonding area. (**H**) WVTR of various films. (**I**) Radar chart comparing characteristic performances. The performance of the ultrathin stretchable ACF is presented for each indicator, with maximum and minimum indices for each characteristic shown in the chart. Legend colors correspond to the colors in the chart. Film thickness is estimated from figures in the literature if not explicitly stated in the text. For WVTR not reported in the literature is indicated as N/R. For bonding pressure, unspecified values are assumed to be low values. All data are presented as means ± SD (*n* = 3), with error bars indicating the SD. Yellow lines (wire resistance) indicate SD bounds for the standalone parylene-based Cr/Au films.

The adhesion performance of the ultrathin stretchable ACF was evaluated using a wet-volatile process. Ethanol, which is highly volatile and applicable to biological systems, was used as the volatile liquid (*34*). The bonding forces measured in the T-peel test (90° peel from the bonding interface) and the shear strength test were comparable for samples bonded using the wet-volatile process and those bonded with applied pressure (8.5 kPa) (Fig. 3A). The bonding force from the wet-volatile process was 11% (T-peel test) and 37% (shear strength test) of the peel force observed for commercial ACF (Fig. 3B). When two 4-μm-thick parylene films were bonded using the ultrathin stretchable ACF and the wet-volatile process, they fractured within the monolayer parylene film region before the bonded section failed when subjected to shear strength force (Fig. 3C and movie S2). These results confirm that the combination of the ultrathin stretchable ACF and the wet-volatile process delivers the high tensile strength required to withstand tensile forces generated under stretching conditions. Simultaneously, the relatively weak 90° peel adhesion is advantageous for intentional detachment, such as device replacement or relocation. The time required for bonding force establishment by ethanol volatilization in the wet-volatile process was evaluated by measuring the time-dependent change in bonding force using the shear strength test during the bonding of gas-impermeable polyimide (PI) films using the ultrathin stretchable ACF (fig. S9). The bonding force achieved after a 30-min volatilization time was equivalent to that achieved after longer volatilization times in Fig. 3B. It was confirmed that 87% of the bonding force achieved after 30 min was established after 15 min of volatilization, and 46% was established in 1 min. This characteristic, allowing weak bonding to initiate quickly and gradually solidify into robust bonding even on highly gas-impermeable films, is a useful property for joining fragile thin-film electronics. Furthermore, it is expected that ethanol volatilizes more quickly on substrates with higher gas permeability.

The through-thickness electrical resistance was measured during stretching (Fig. 3D). The electrical resistance change across 125 to 560% strain was ±11% compared with the unstretched state, confirming stable conductivity under stretching. The electrical stability under biaxial stretching was also evaluated (fig. S10), confirming a maximum stretchability of 200%. The resistance changes under 50 to 200% biaxial strain were ∼−4%, demonstrating stable conductivity regardless of the degree of stretching. In addition, a thicker ACF (using a 12 wt % SEBS solution) maintained low resistance even at 1000% strain (fig. S11). Next, electrical resistance was measured in the relaxed state after 1000 stretching cycles at various strains (Fig. 3E). The resistance change across 125 to 250% strain was −12% compared with the unstretched state. This is comparable to the resistance change during stretching, confirming that the ultrathin stretchable ACF maintains stable conductivity even after repeated mechanical deformation. The electrical stability after 1000 cycles of 250% strain at increased stretching speeds of 500 and 1000 mm/min was measured (fig. S12). The resistance change observed with the increase in stretching speed was 18 to 19%. Critically, the resistance values at 500 and 1000 mm/min were comparable, indicating robust stability at high speeds. These results confirm that the ultrathin stretchable ACF maintains high electrical stability and durability under stretching, demonstrating its effectiveness in stretching environments.

The anisotropic electrical properties of the ultrathin stretchable ACF were confirmed by measuring the current flow upon applying a voltage across the bonded parylene-based Cr/Au films. This test specifically assessed both the through-thickness conductivity between overlapping electrodes and the in-plane insulation between electrodes separated by 200 μm (Fig. 3F). A linear change in current with respect to applied voltage was observed between the overlapping electrodes (electrodes 1 and 3 in Fig. 3F), whereas complete insulation was maintained between the electrodes separated by 200 μm (electrodes 1 and 4 in Fig. 3F). The dependence of the electrical resistance on the bonding area was also measured (Fig. 3G). The electrical resistance increased as the bonding area decreased, reaching 8.6 Ω (including the wiring resistance) at a bonding area of 0.06 mm^2^. These results indicate that the ultrathin stretchable ACF enables bonding at a resolution of a few hundred micrometers. Furthermore, the electrical resistance stability of the parylene-based Cr/Au films bonded with the ultrathin stretchable ACF during storage in air was confirmed (fig. S13). The rate of change from the initial resistance after 30 days was 3%, confirming that the ultrathin stretchable ACF maintains stable resistance with minimal effects from atmospheric oxidation. The stability of the ultrathin stretchable ACF in contact with artificial sweat (81301, Muto Pure Chemicals) was evaluated under accelerated conditions of high temperature and high humidity [40°C/90% relative humidity (RH)] (fig. S14). After 24 hours, the resistance change was 1.6 ± 4.9%, and no notable morphological changes were observed via digital microscopic observation. Because AgNWs are generally susceptible to degradation by air and sweat, future practical applications aiming for both enhanced biocompatibility and stability may consider substituting the AgNWs with gold nanowires. The electrical stability was evaluated by a temperature cycling test (−20°C/+85°C, 10-min hold at each temperature, 20 cycles) and across a temperature range of −20° to +110°C (figs. S15 and S16). The sample showed a resistance change of −3.9 ± 3.2% after the temperature cycling test. Furthermore, when referenced to 25°C, the resistance changes across the temperature range from −20° to +110°C was ∼−2 to +6%.

The WVTR of the ultrathin stretchable ACF was measured by covering a water-filled bottle with the film and measuring the water loss (Fig. 3H), yielding a value of 1230 g/m^2^ per day. This value is comparable to 1350 g/m^2^ per day measured for an SEBS-only film (3 wt % concentration and without AgNWs), suggesting that the addition of AgNWs has a limited impact. For comparison, a medical-use skin adhesion film (Tegaderm Transparent Film Roll 16004, 3M) has a WVTR of 180 g/m^2^ per day. The ACF using 12 wt % SEBS showed a decrease in WVTR owing to the increased film thickness, becoming comparable to that of the medical skin adhesion film (table S1). These results indicate that the ultrathin stretchable ACF has a higher WVTR than films intended for on-skin use, demonstrating properties suitable for bonding wearable devices.

In summary, the ultrathin stretchable ACF exhibits superior performance compared with previous studies in terms of film thickness (∼0.3 μm), conductive stretchability (>500%), WVTR (1200 g/m^2^ per day), bonding temperature (∼25°C, room temperature), and bonding pressure (0 MPa) (*29*, *30*, *32*, *33*, *35*) (Fig. 3I and table S2).

### Demonstration of the ultrathin stretchable ACF applications

**Fig. 4.**
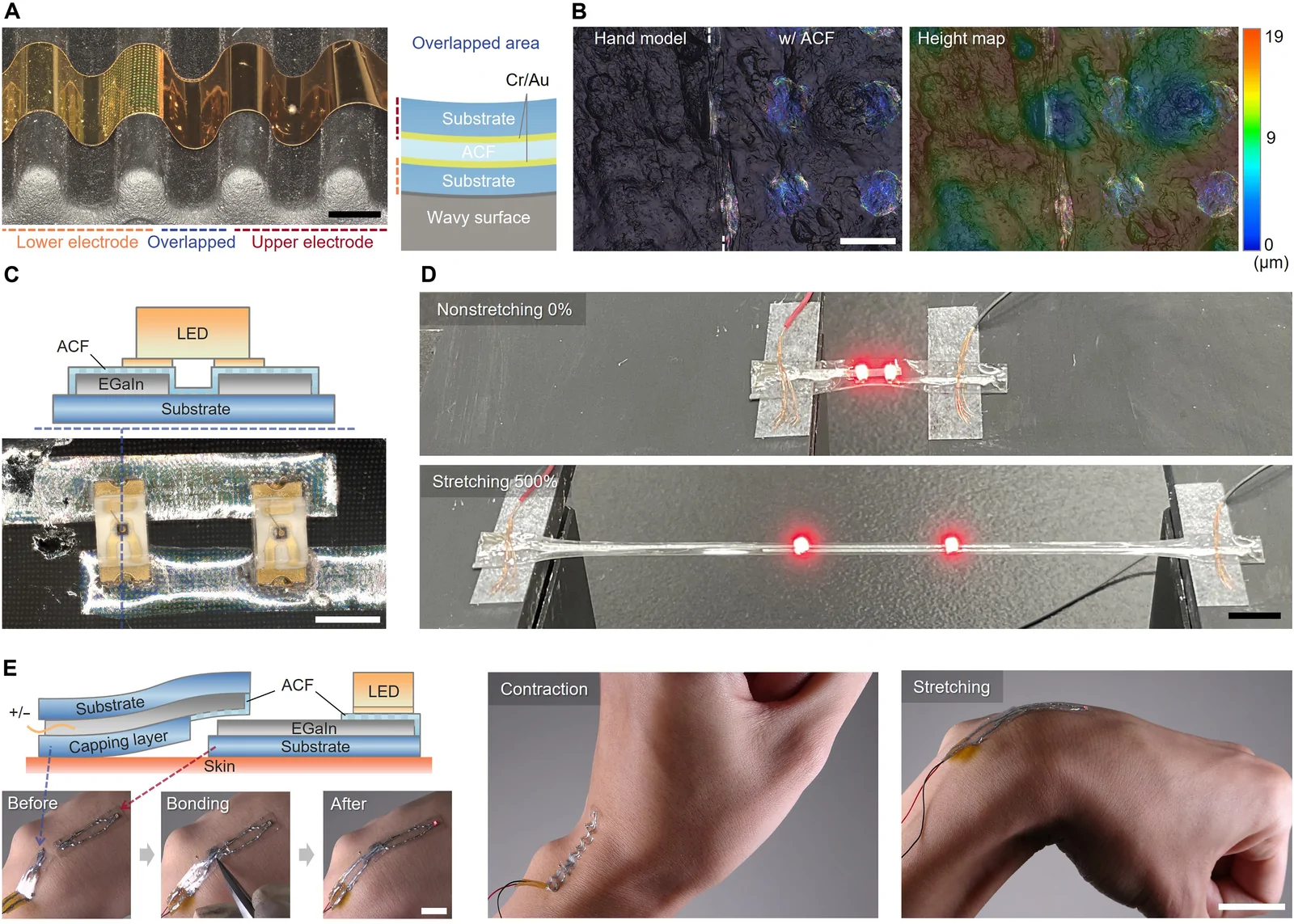
Demonstrations using the ultrathin stretchable ACF. (**A**) Photographs of the ultrathin stretchable ACF-bonded parylene-based Cr/Au films conforming to a wavy surface. (Left) Schematic cross section of the ultrathin stretchable ACF-bonded region; (right) dotted lines indicate the corresponding areas across the images. Scale bar, 1 mm. (**B**) Evaluation of the ultrathin stretchable ACF bonded onto a hand model with an uneven surface. (Left) Optical image showing the left region without ACF and the right region with the ultrathin stretchable ACF. Scale bar, 50 μm. (Right) Height map where color represents the height distribution. (**C**) Device structure for the LED-integrated stretchable demonstration. (Top) Schematic cross section of an LED bonded with the ultrathin stretchable ACF onto EGaIn stretchable wiring (the cross section corresponds to the dashed line); (bottom) top-view photograph. Scale bar: 1 mm. (**D**) Photographs demonstrating the stretchability of the LED-integrated substrate, (top) the relaxed state, (bottom) under 500% strain. Scale bar, 5 mm. (**E**) Schematic and images showing the bonding between stretchable devices on skin, including images of the device in its relaxed and stretched states. Scale bars, 10 mm (left) and 20 mm (right).

The ultrathin stretchable ACF retained the flexibility of the bonded structures (Fig. 4A). Parylene-based Cr/Au films [Cr/Au (3.5/100 nm) on 2-μm-thick parylene] were bonded with the ultrathin stretchable ACF to observe their conformability to a wavy surface with a radius of curvature of 500 μm. The bonded film exhibited a wavy surface without delamination, even in the overlapping bonded region. Its nanoscale, soft, and stretchable properties enable bonding without compromising film flexibility.

The applicability of the ultrathin stretchable ACF to microrough surfaces was also demonstrated (Fig. 4B). The ultrathin stretchable ACF was integrated onto a hand-modeled surface using a wet-volatile process. Surface roughness (arithmetic mean height) was comparable before (hand model: 2.24 μm) and after (with ultrathin stretchable ACF: 2.27 μm) integration of the ultrathin stretchable ACF. In addition, the height map confirmed that the original surface topology of the hand model remained clearly observable even after integration of the ultrathin stretchable ACF. This demonstrates that despite the ultrathin stretchable ACF’s flat film shape, its high flexibility and the damage-free wet-volatile process enable it to conform and adhere closely even to surfaces with micrometer-scale roughness.

The ultrathin stretchable ACF was used to integrate rigid parts into a stretchable device (Fig. 4C). A stretchable wiring substrate was fabricated on a thick SEBS film using EGaIn with a pitch of 500 μm. The stretchable wiring was covered with the ultrathin stretchable ACF, and the LEDs were lightly pressed onto it for bonding. Even after 500% strain cycles while supplying voltage to the circuit, the conductivity and insulation between the wires were stable, and the LEDs operated reliably (Fig. 4D and movie S3).

Bonding between stretchable devices on the skin using the ultrathin stretchable ACF was demonstrated (Fig. 4E and movie S4). A stretchable connector device with power input wiring was connected to a premounted stretchable device composed of EGaIn stretchable wiring and an integrated LED on the wrist using the skin as the substrate, using the ultrathin stretchable ACF. Bonding was achieved by gentle contact at room temperature. The conductive connection was maintained, and the LED operated reliably even under contraction and stretching induced by multiple wrist movements.

Signal stability of a strain sensor fabricated on a mesh substrate and interconnected using the ultrathin stretchable ACF was evaluated on skin (fig. S17). A nonstretchable control sample was prepared by applying PI tape over the ultrathin stretchable ACF junction to prevent stretching. The strain sensor interconnected with the ultrathin stretchable ACF maintained stable signal acquisition throughout repeated wrist bending and resting cycles (normalized resistance of 1.3 after bending). In contrast, the control sample exhibited an irreversible resistance increase of 28 after multiple bending cycles, resulting in degraded signal stability.

## DISCUSSION

An ultrathin stretchable ACF was fabricated by spraying metal nanowires over a mask to form a conductive pattern, followed by infiltration of an elastomer solution. Conventional alignment methods for creating equally spaced circular conductive regions are often limited by complex procedures and constraints related to conductive particle materials (*32*, *36*). Our method enabled the fabrication of ACF structures with complex conductive patterns by simply changing the mask design. Modifying the pattern based on device location allows adjustment of the resolution and localized control of the conductive properties. Furthermore, ACF structures with similar architectures can be fabricated even if the metal nanowire and elastomer materials are changed. Although it is necessary to optimize the fabrication conditions for each pattern and material, this technique demonstrates the potential for creating ACF with previously unrealized functionalities.

The AgNW/SEBS structure, which deformed with the film, simultaneously achieved a high WVTR owing to its ∼300-nm thickness and stable conductivity beyond 500% strain, exhibiting mechanical properties close to those of a monolayer SEBS film. Furthermore, we demonstrated that film bonding is possible under damage-free conditions using a wet-volatile process or by applying a small load. SEBS and AgNWs have been applied to various body parts, such as the skin and heart (*37*–*40*). These properties suggest that the ultrathin stretchable ACF enables interconnections between devices on diverse surfaces. Conventional methods that involve heating, pressure, or UV irradiation pose challenges for bonding on uneven, soft, or stimulation-sensitive surfaces, such as biological tissues. Such applications have previously necessitated external preprocessing of device interconnections before mounting. The high stretchability, exclusive use of biocompatible materials, and damage-free bonding process of the ultrathin stretchable ACF allow bonding on the aforementioned surfaces. This capability enables the repair, modification, or expansion of device functions through reversible connections and disconnections in real-world applications.

The wet-volatile process enables damage-free bonding; however, it requires relatively long processing times on gas-impermeable substrates due to its reliance on ethanol volatilization. Furthermore, the bonding strength is constrained by the intrinsic material properties of the SEBS matrix (table S2). Accordingly, selecting an appropriate bonding mechanism by adjusting the resin material based on the interconnection target is crucial. For heat-resistant substrates such as printed circuit boards or flexible circuits, a hot-press method using heat and pressure enables robust adhesion. For applications involving UV-stable substrates that do not involve sensitive surfaces such as biological tissues, a UV curing method can be used. Conversely, the wet-volatile process remains the most effective solution for integrating electronics onto sensitive surfaces where damage-free bonding is mandatory.

The functional importance of nanoscale thinness in this study is dictated by two primary factors: enhanced flexibility and increased gas permeability. The bending stiffness of a film is proportional to the third-degree term of its thickness. Therefore, the ultrathin design reduces stiffness and improves flexibility, which is crucial for achieving intimate conformal interfaces. Furthermore, although SEBS provides the necessary stretchability and adhesion, it is intrinsically a material with low gas permeability. However, sufficiently reducing the film thickness substantially enhances gas permeability (table S1). The combination of the current transfer method and high mechanical stretchability adequately addresses potential fragility during the manufacturing process. However, for large-scale productization, there is a potential need to explore simpler and more robust integration methods.

The demonstrated 200% biaxial stretchability is a critical attribute for ultrathin stretchable electronics designed to achieve conformal contact by adhering to the complex topography of the skin. Unlike conventional on-skin devices, these ultrathin systems must accommodate strains exceeding 50 to 80%, the typical maximum elongation of human skin, to allow for additional deformations occurring during the process of achieving high structural adhesion (*41*, *42*). Furthermore, in the field of soft robotics, there is an increasing demand for circuits exhibiting stretchability exceeding several hundred percent to support advancements in biomimicry and highly compliant soft actuators (*16*, *43*). Crucially, maintaining a stretchability margin well beyond these practical requirements is essential for ensuring long-term cyclic durability. By operating the device well below its ultimate strain limit, microstructural fatigue accumulation is minimized, effectively suppressing electrical and mechanical degradation during repetitive movements. Consequently, the stretchability of the ACF technology presented in this study is ideally positioned as an advanced, durable interconnection method for both on-skin and soft-robotic systems.

The high-density pixelated arrays are an important direction for further advancing ACF-based wearable systems. To achieve higher density integration, transitioning from mask-based patterning to self-assembly techniques represents an effective strategy for further scaling down feature sizes. For example, the self-organization of AgNWs to form micrometer-diameter rings (*44*) offers a promising pathway to refine our dot-pattern resolution. In addition, the ultrathin stretchable ACF could serve as an interconnection technology for high-fidelity signal acquisition. Integrating our ACF with specialized low-noise sensors (*45*, *46*) and/or robust, environmentally shielded conductor technologies (*47*, *48*) would be a promising trajectory for the development of high-performance signal acquisition systems.

In summary, the ultrathin stretchable ACF developed in this study simultaneously achieved a nanoscale structure (high WVTR) and high mechanical and electrical stretchability through its AgNW/SEBS structure. The structure was fabricated using a simple and highly scalable process of infiltrating elastomers into the metal nanowire structure. We also demonstrated that a damage-free bonding method using the wet-volatile process enables the conformal bonding of ultrathin stretchable ACF to targets without requiring heating or pressurization. This high-performance ACF is nanoscale, soft, and highly stretchable and has a high WVTR with damage-free bonding conditions, thereby contributing to the further functional enhancement of stretchable electronics. Advancements in interconnection technology are expected to contribute to the development of stretchable electronic devices and applications.

## MATERIALS AND METHODS

### Ultrathin stretchable ACF fabrication

To fabricate a weakly adhesive mask for patterning AgNWs, a 25-μm-thick PI film (UPILEX, UBE) was cleaned with oxygen plasma using a plasma cleaner (PC-300, Samco). A 3 wt % SEBS (Tuftec H1221, Asahi Kasei) solution in toluene was spin coated onto the treated PI film and annealed at 110°C for 10 min to form an adhesive layer. The film was then adhered side-down to a glass slide using water. The weakly adhesive mask was finalized by perforating 40-μm-diameter holes with a 40-μm pitch using a UV laser marker (MD-U1000, KEYENCE).

Glass slides were cleaned using a plasma cleaner. A sacrificial layer was formed by spin-coating PSS [poly(sodium 4-styrenesulfonate)] [30 mg/ml; weight-average molecular weight (*M*_w_) = 1,000,000, Sigma-Aldrich] onto a glass slide and annealing at 110°C for several minutes. The weakly adhered mask was lightly pressed and adhered onto the glass slide containing the sacrificial layer. The glass slide was placed on a hot plate and heated to 110°C. The AgNW ink (AW030, Zhejiang Kechuang Advanced Materials Technology) [diameter = 25 to 35 nm, length = 10 to 30 μm, 1 wt % AgNWs in isopropyl alcohol (IPA)] was diluted with IPA to 0.1 wt %. This 2 ml of ink was spray coated from 10 cm above the mask at an atomization pressure of 0.3 MPa. The substrate was then annealed for 5 min. The mask was subsequently peeled from the glass slide. A 3 wt % SEBS solution was then spin coated and annealed at 110°C for 5 min. The fabricated AgNW/SEBS film was peeled from the glass slide by infiltrating water and dissolving the sacrificial layer to create an ultrathin stretchable ACF. The ultrathin stretchable ACF was wetted with ethanol (mass fraction ≥99.5%, FUJIFILM Wako Pure Chemical) and applied to each target surface. Bonding was then performed using the wet-volatile process by leaving the assembly at room temperature until the ethanol had completely volatilized.

### Fabrication of parylene-based Cr/Au films

A 2-μm-thick parylene film (diX-SR, Daisan Kasei) was deposited on a glass slide coated with a fluorinated polymer layer (Novec 1700, 3M) via chemical vapor deposition using a deposition system (PDS 2010, Kisco). To improve adhesion between the parylene film and Au, Cr (3.5 nm) was deposited, followed by Au deposition to a thickness of 100 nm. The resulting film was patterned by cutting it with a UV laser marker and subsequently peeling it from the glass slide.

### Fabrication of films using deposited silver

A 500-nm-thick Ag film was deposited onto a glass slide using a vacuum evaporator. The glass slide was coated with a sacrificial layer (prepared using the same method as that used for ultrathin stretchable ACF fabrication) and covered with a weakly adhesive mask. After deposition, the mask was removed from the glass slide. SEBS (3 wt %), at the same concentration as that used for the comparison ACF, was spin coated onto the deposited silver film and annealed at 110°C for 5 min. The film containing the deposited silver was obtained by peeling it from the glass slide after infiltrating water and dissolving the sacrificial layer.

### Cross-sectional and surface SEM observations

The specimen for cross-sectional observation was prepared using an ion beam milling system (EM TIC 3X, Leica). SEM images and EDX mapping were obtained using an SEM instrument (Quattro S, Thermo Fisher Scientific).

Surface SEM images were obtained at an angle of 45° using a tabletop SEM (VHX-D510, KEYENCE).

### Observation of AgNW structure change during stretching

A thick SEBS film (∼100 μm) was prepared by pouring 19 ml of 13 wt % SEBS solution into a 150-mm-diameter glass mold and allowing the solvent to volatilize in a room-temperature chamber. An ACF with 3 wt % SEBS was adhered to this film. The sample was fixed to a tensile-compression stage with a base length of 5 mm. The deformation during stretching was observed using a digital microscope (VHX-7100; KEYENCE). In addition, the sample state was observed before and after 1000 stretching cycles at 500% strain at a speed of 100 mm/min.

### Measurement of the electrical resistance of ACFs at different SEBS solution concentrations

Films were fabricated with SEBS solution concentrations of 3, 12, and 18 wt % using the same fabrication process as that used for the ACF. For each film, 5-mm-wide parylene-based Cr/Au films were bonded with a bonding area of 5 mm by 5 mm. A digital multimeter (34465A, Keysight Technologies) was connected to both ends of the parylene-based Cr/Au films, and the electrical resistance was measured using the four-terminal method. Three experiments were conducted under each condition.

### Analysis of the shape change of deposited silver and AgNWs during stretching

The deposited silver sample and the AgNW sample were adhered to a 125-μm-thick PI film (UPILEX, UBE). To prevent fracture due to stress concentration between the PI and the ultrathin stretchable ACF, the samples were cut using a UV laser marker to an initial length of 4 mm and fixed to a tensile-compression stage (fig. S18). The deformation state during stretching was photographed under each condition using a digital microscope. From these images, the distance between the centers of the two conductive particles in the stretching direction and the ratio of the horizontal to vertical widths of the conductive particles were calculated. Three measurements were taken for the center-to-center distance, and six measurements were taken for the horizontal-width/vertical-width ratio under each condition. For ideal SEBS deformation, the distance between two conductive particles was assumed to be equivalent to the strain rate. The horizontal-width/vertical-width ratio was calculated assuming uniform contraction in the width and thickness directions during film stretching.

### Measurement of maximum fracture force for films using deposited silver and the ultrathin stretchable ACF

A monolayer SEBS film adjusted to a thickness similar to that of the ultrathin stretchable ACF, a film using deposited silver, and the ultrathin stretchable ACF using 3 wt % SEBS were fixed to a tensile-shear tester (EZ-LX, Shimadzu) with a base length of 4 mm, similar to the method described in “Analysis of the shape change of deposited silver and AgNWs during stretching.” The film was stretched at a rate of 1 mm/min until complete fracture occurred, and the force generated at that point was measured. After a complete fracture, the measured force was 0 N, and the maximum force generated before fracture was recorded as the maximum fracture force. Three experiments were conducted under each condition.

### Measurement of bonding strength

For the shear strength test, 2-mm-wide, 12.5-μm-thick PI films (UPILEX, UBE) were bonded under each bonding condition with a bonding area of 2 mm by 2 mm. The force required to pull the sample in the 180° direction at a speed of 1 mm/min was measured using a tensile-shear tester. For the T-peel test, 4-μm-thick parylene films fabricated using the same method as that used for the parylene-based Cr/Au films were cut to a width of 2 mm and bonded such that the bonding width was 2 mm and the length was ≥2 mm. The films were fixed to a T-shaped tensile tester, and the force required to pull the sample at 1 mm/min was measured. For the load-free ultrathin stretchable ACF and commercial ACF (9703, 3M), bonding was performed using the wet-volatile process. For consistency, ethanol was allowed to volatilize by leaving the samples at room temperature for ≥30 min. For the loaded ultrathin stretchable ACF condition, the ultrathin stretchable ACF and the films to be bonded were stacked and sandwiched between 24-mm-square cover glasses. A 500-g weight was placed on top, applying a pressure of 8.5 kPa. Bonding was performed by leaving the assembly at room temperature for ≥30 min. Three tests were conducted for each condition. For the shear strength test, the maximum force measured in each test was averaged. For the T-peel test, the force over a 2-mm range from the position where the force first reached its peak was averaged. The force during the shear strength test was measured for 12.5-μm-thick PI films bonded by the wet-volatile process, with volatilization times ranging from 1 to 30 min.

Furthermore, 4-μm-thick parylene films, fabricated using the same method as the parylene-based Cr/Au films, were bonded using the ultrathin stretchable ACF with a bonding area of 2 mm by 2 mm, and the state during stretching on a tensile-compression stage was observed with a digital microscope.

### Measurement of conductivity during stretching

The sample was fixed to a tensile-compression stage with a base length of 4 mm, similar to the configuration described in “Analysis of the shape change of deposited silver and AgNWs during stretching.” Stretching was performed by moving the tensile-compression stage. For conductivity measurements during simple stretching, 2-mm-wide parylene-based Cr/Au films were bonded with a bonding area of 2 mm by 2 mm using an ultrathin stretchable ACF stretched to a specific strain rate by the stage. A digital multimeter was connected to both ends of the parylene-based Cr/Au films, and the electrical resistance was measured using the four-terminal method. The ultrathin stretchable ACF was fixed within the 8-mm-diameter aperture of a silicone rubber sheet (Ecoflex 00-30, Smooth-On) and stretched by biaxially extending the sheet. The electrical resistance was then measured similarly while the ultrathin stretchable ACF was stretched. For the cycle test, the resistance was measured under the same conditions as those used for simple stretching. The ultrathin stretchable ACF was then subjected to 1000 stretching cycles at a specific strain rate and a speed of 100 mm/min, after which the strain returned to 0%. The electrical resistance was also measured in the relaxed state after 1000 stretching cycles at 250% strain performed at higher speeds of 500 and 1000 mm/min. The electrical resistance of the overlapping parylene-based Cr/Au films used in the experiment was measured and defined as the wire resistance. Three tests were conducted under each condition.

### Measurement of electrical characteristics (insulation, area, and long-term stability)

Parylene-based Cr/Au films were used for all electrical measurements, and three experiments were conducted under each condition. To evaluate insulation at narrow pitch, films were arranged with a 200-μm separation and bonded with the ultrathin stretchable ACF. A source meter (2400, Keithley Instruments) was used to apply a voltage of −0.5 to 0.5 V, and the resulting current was measured to confirm electrical insulation. To determine conductivity as a function of bonding area, the films were cut to various widths (0.25, 0.5, 0.75, and 1 mm) and bonded to form square bonding areas. The resistance was measured using a digital multimeter, and the electrical resistance of the connecting wiring was measured and defined as the wiring resistance subtracted from the total resistance. For long-term stability, the films (5 mm wide) were bonded (bonding area of 5 mm by 5 mm) using ultrathin stretchable ACF (3 wt % SEBS concentration). These samples were stored in ambient atmosphere for 30 days, and the resistance was periodically measured using a digital multimeter connected to both ends of the films. The resistance change of 2-mm-wide parylene-based Cr/Au films, bonded with the ultrathin stretchable ACF with a bonding area of 2 mm by 2 mm, was also measured after the samples were covered with nonwoven fabric soaked in artificial sweat (81301, Muto Pure Chemicals) and stored for 24 hours at 40°C/90% RH in an environmental test chamber (SH-222, ESPEC). In addition, the ultrathin stretchable ACF was similarly covered with artificial sweat-soaked nonwoven fabric and stored under the same conditions, followed by observation using a digital microscope. The electrical resistance change of similarly bonded 2-mm-wide parylene-based Cr/Au films was measured after a temperature cycling test (−20°C/+85°C, 10-min hold at each temperature, 20 cycles), and the resistance was measured across a temperature range from −20° to +110°C. Six samples were tested for stability against artificial sweat and temperature cycles.

### Measurement of WVTR

Ten milliliters of pure water was placed in 13.5-ml bottles with an inner diameter of 14.5 mm. For the open condition, the bottles were capped with aluminum foil containing a 5-mm-diameter hole. For each film condition, the bottles were capped with aluminum foil with the film covering the 5-mm hole. A medical-use skin adhesion film (Tegaderm Transparent Film Roll 16004, 3M) was used as a control. SEBS films were prepared by spin-coating SEBS onto a glass slide with a sacrificial layer deposited to achieve a film thickness similar to that of the ultrathin stretchable ACF. Each capped sample was placed in a desiccator at 21°C and 30% humidity, and the water loss was calculated by periodically measuring the total weight of the samples. Three experiments were conducted under each condition.

### Conformability on complex surfaces

Conformability was evaluated by mounting the film on a wavy surface. For this test, 2-mm-wide parylene-based Cr/Au films were bonded using the ultrathin stretchable ACF. The bonded sample was mounted on a wavy surface with a radius of curvature of 500 μm, which was fabricated using 3D-printed resin (Black Resin, Formlabs, Form4). Samples were observed from the surface at an angle using a digital microscope. For mounting on a hand model, the ultrathin stretchable ACF was integrated onto the surface of a 3D-printed resin hand model using the wet-volatile process. The sample was observed from the surface using a digital microscope, and height mapping was performed using the microscope’s measurement function.

### Stretching of the LED-integrated substrate using the ultrathin stretchable ACF and on-skin bonding

EGaIn [75.5% Ga/24.5% In, ≥99.99% trace metals basis (495425), Sigma-Aldrich] was spray coated onto a thick SEBS substrate using a mask with 1-mm-width patterns and a 500-μm pitch. An ultrathin stretchable ACF was bonded to cover the EGaIn wires. The LEDs (LTST-C190KRKT, Lite-On) were lightly pressed onto the ultrathin stretchable ACF for bonding. Strain was applied to the entire substrate while the voltage was supplied from a constant-voltage source.

A stretchable device fabricated under similar conditions was integrated onto the skin of the wrist using a medical-use skin adhesion film. One end of the stretchable connector device (featuring similar EGaIn wiring) was covered with the ACF, whereas the other end was connected to a constant-voltage source, with the remaining part covered by a medical-use skin adhesion film. Bonding was achieved by gently pressing the ACF-covered region onto the stretchable device.

### Wrist bending measurement with the ultrathin stretchable ACF-interconnected mesh sensor

A 15 wt % polyurethane (PU) solution was prepared by diluting a commercial PU solution (Rezamin M-8115LP, 30 wt %, Dainichiseika Color & Chemicals Mfg.) with *N*,*N*′-dimethylformamide (FUJIFILM Wako Pure Chemical). A 1-ml aliquot of the solution was electrospun (NANON-03, MECC) using a 22-gauge needle at an applied voltage of 25 kV, a tip-to-collector distance of 13.5 cm, and a feed rate of 1.0 ml/hour to fabricate a PU mesh sheet. A mesh substrate was prepared by blow spinning a 20 wt % SEBS solution onto the PU mesh sheet from a distance of 10 cm using nitrogen gas at 0.3 MPa. A strain sensor was subsequently fabricated by spray coating 3 ml of a 0.1 wt % AgNW dispersion onto the mesh substrate. The strain sensor was connected to the wiring on the mesh substrate on the wrist using the ultrathin stretchable ACF, and the resistance during wrist bending and resting cycles was measured using a digital multimeter. As a control condition, the junction bonded with the ultrathin stretchable ACF was immobilized by applying PI tape (1030-01, AS ONE) over the bond, and the resistance was measured in the same manner.
