# Supplementary material for: Gas-permeable and stretchable nanoscale anisotropic conductive film for room-temperature, pressure-free interconnections
Source: Sci Adv. 2026 Sep 18;12(38):eaeh6203. doi: 10.1126/sciadv.aeh6203 (PMC13588162; doi:10.1126/sciadv.aeh6203)
Supplement: Supplementary file 1 [file sciadv.aeh6203_sm.pdf]

Science Advances
AAAS

Supplementary Materials for

Gas-permeable and stretchable nanoscale anisotropic conductive film for room-temperature, pressure-free interconnections

Shumpei Katayama *et al.*

Corresponding author: Takao Someya, takao.someya@riken.jp; Sunghoon Lee, sunghoon.lee@riken.jp

Sci. Adv. **12**, eaeh6203 (2026)
DOI: 10.1126/sciadv.aeh6203

The PDF file includes:

Figs. S1 to S18
Tables S1 and S2
Legends for movies S1 to S4

Other Supplementary Material for this manuscript includes the following:

Movies S1 to S4

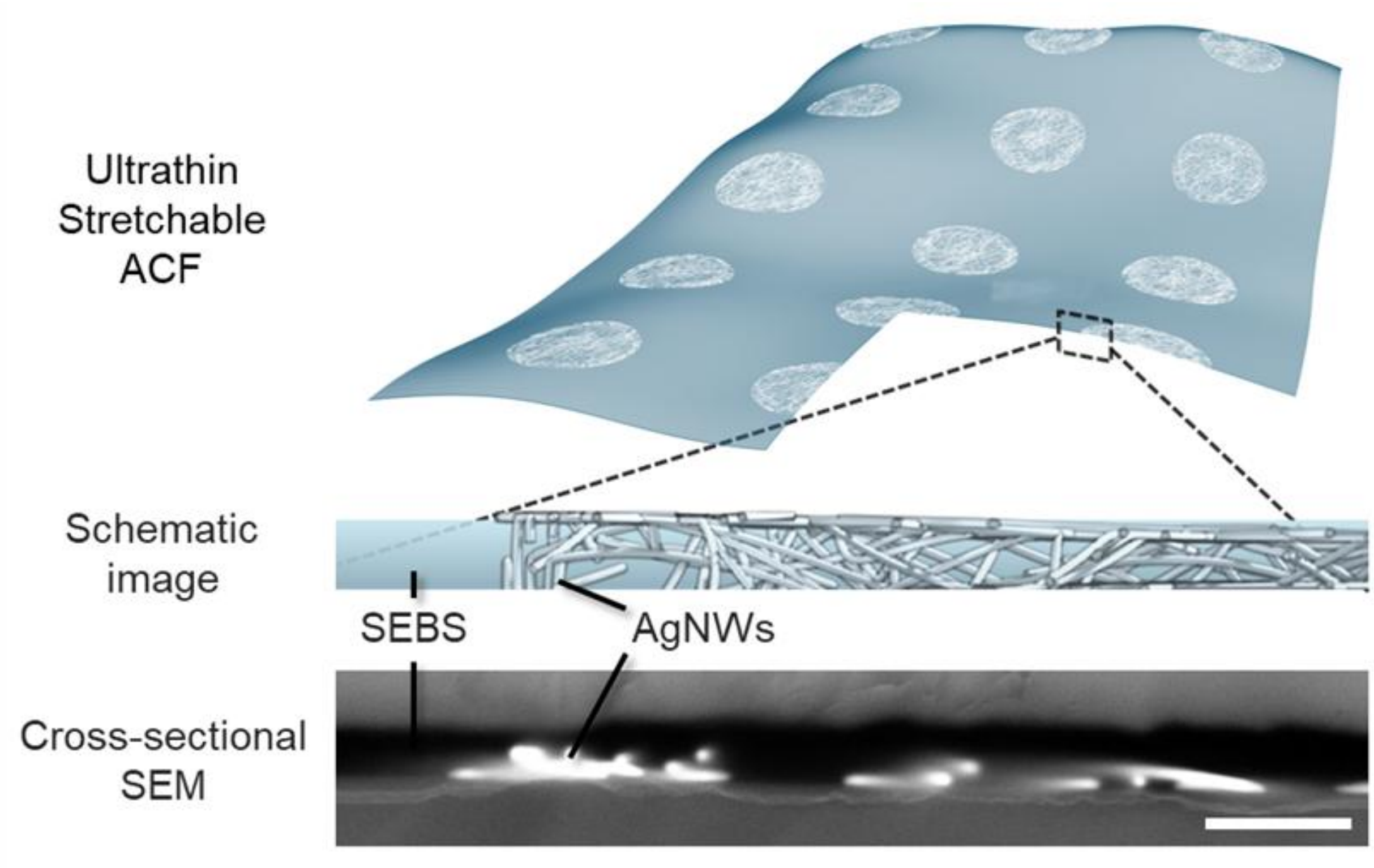

Fig. S1. Cross-sectional Structure of the Ultrathin Stretchable ACF. Schematic illustration of the cross-sectional structure. (Top) Cross-sectional SEM image. (Bottom) Black areas indicate SEBS, and white areas indicate AgNWs. Scale bar: 500 nm.

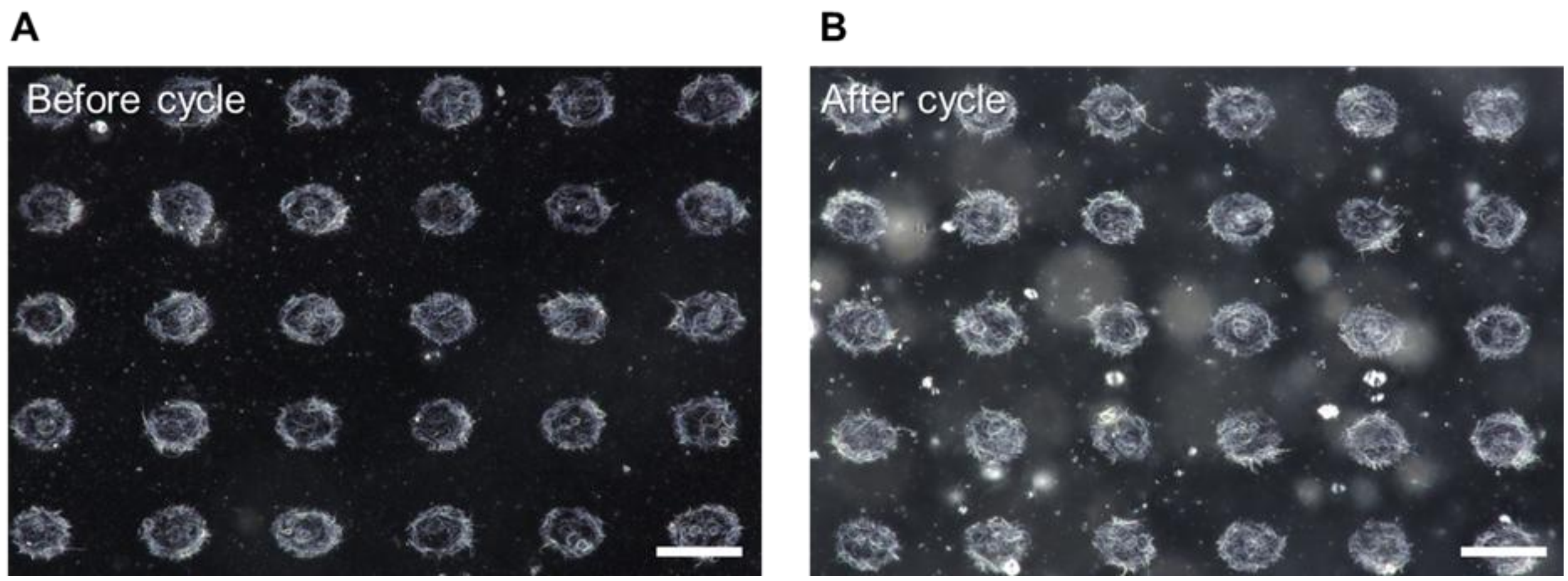

Fig. S2. Optical Images of the Ultrathin Stretchable ACF Before and After Cycles Strain. (A) Top-view optical image of ACF laminated on a thick SEBS film. (B) Top-view optical image of ACF after 1,000 cycles of 500% strain with the load removed. Scale bar: 60 µm.

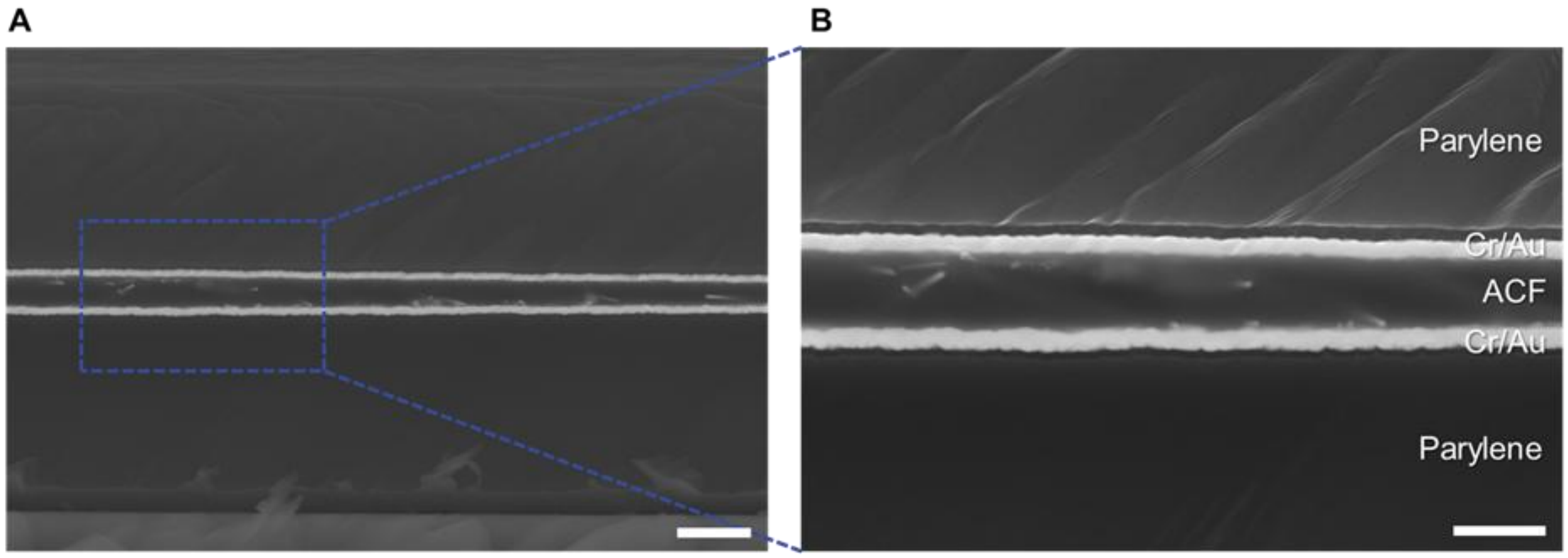

Fig. S3. Cross-sectional SEM Images of a Sample Where Conductive Wiring Films Are Bonded with the Ultrathin Stretchable ACF. (A), (B) Cross-sectional SEM images of two conductive wiring films, which are Cr (3.5 nm)/Au (100 nm) deposited on 2 µm-thick parylene substrates, bonded with ACF via the wet-volatile process. (A) Wide-range cross-sectional view. Scale bar: 1 µm. (B) Magnified cross-sectional view. Scale bar: 500 nm.

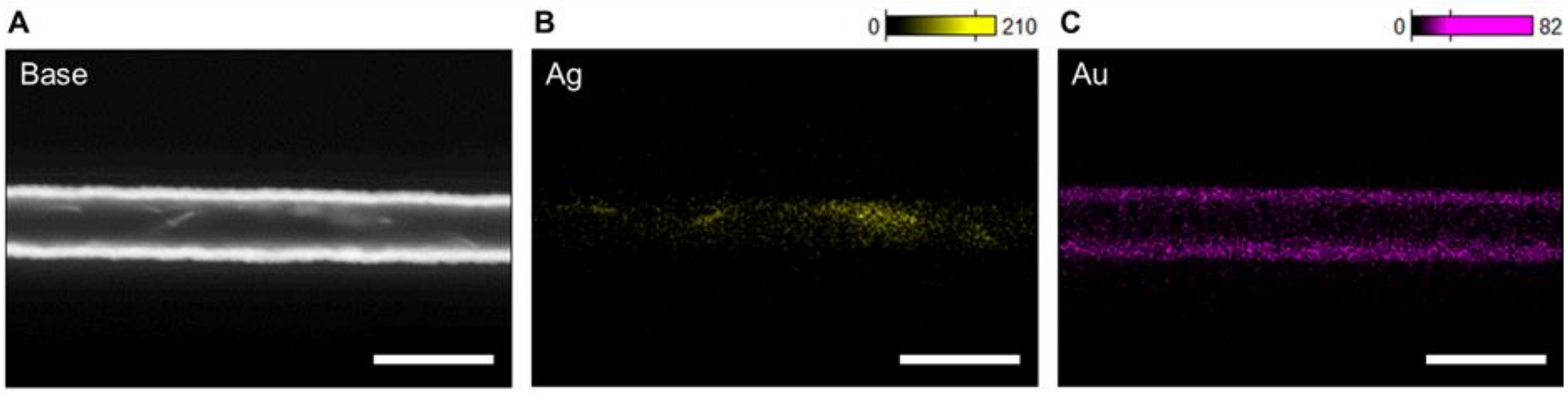

Fig. S4. EDS Mapping of the Cross-section of a Sample Where Conductive Wiring Films Are Bonded with the Ultrathin Stretchable ACF. (A) Cross-sectional SEM image of the bonded sample used for EDS mapping. (B) Ag mapping. (C) Au mapping. Scale bar: 1 μm.

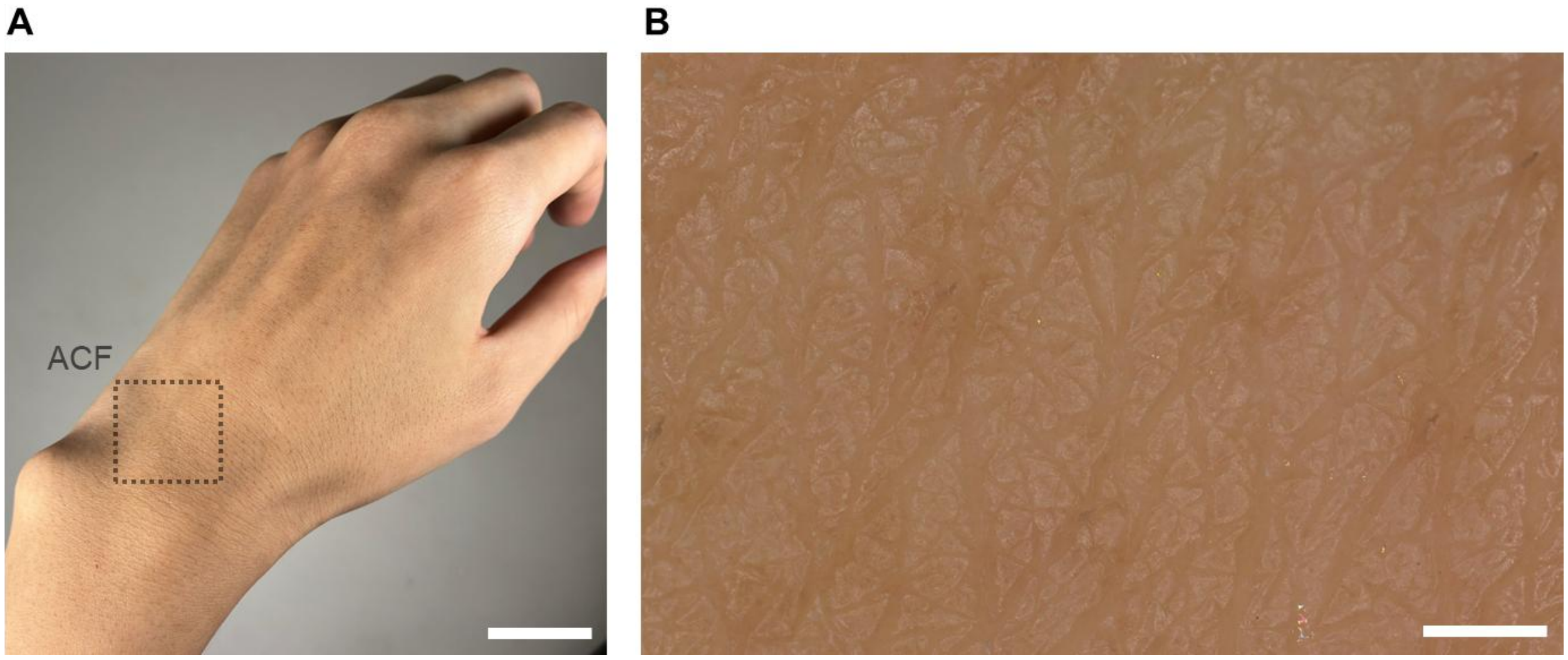

Fig. S5. Optical Images of the Ultrathin Stretchable ACF Integrated onto the Skin. (a) Image of the ACF conformed to the skin after removing the frame from the state shown in Fig. 1E. Scale bar: 10 mm. (b) Top-view optical image of the ultrathin stretchable ACF integrated onto the wrist skin via the wet-volatile process. Scale bar: 1 mm.

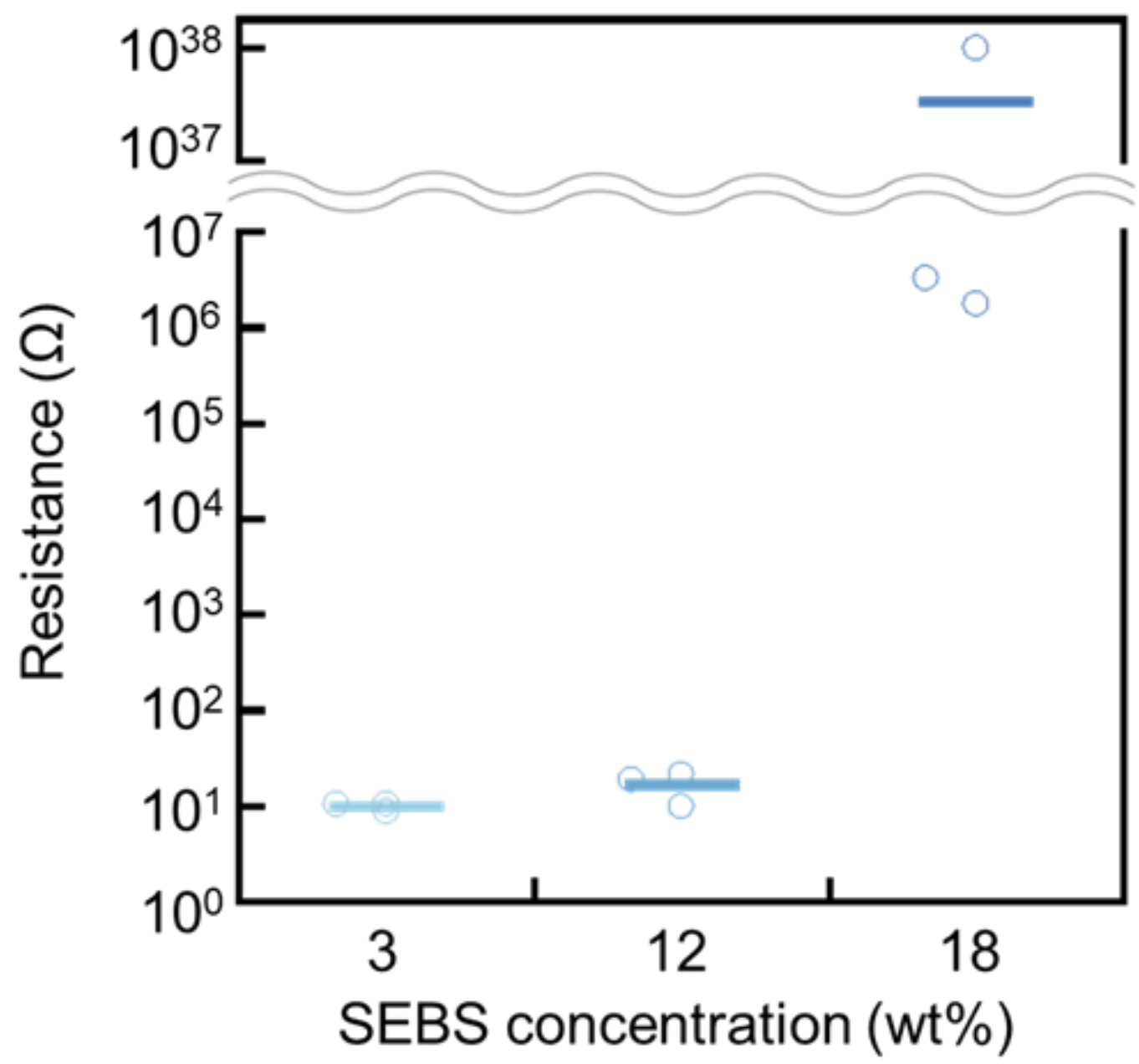

Fig. S6. Through-thickness Electrical Resistance of ACF as a Function of SEBS Concentration. Through-thickness electrical resistance of ACF with varying SEBS concentrations (3, 12, and 18 wt%) used during fabrication. Individual data points represent the resistance values, and the line indicates the mean of three samples (n = 3).

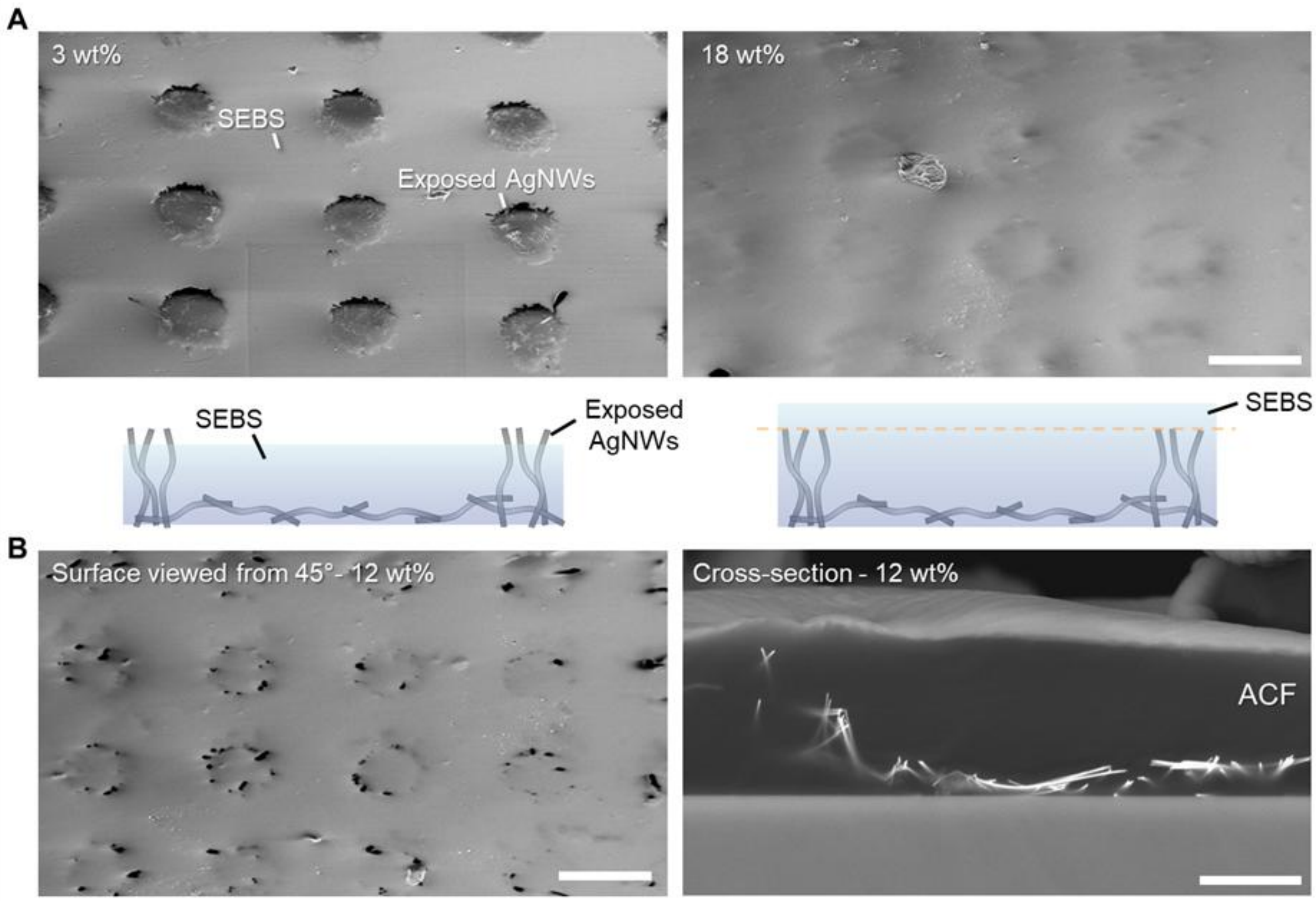

Fig. S7. Surface SEM Images and Cross-sectional SEM. (A) Surface SEM images (viewed at a 45° tilt) and schematic illustrations of the cross-section for 3 wt% and 18 wt% SEBS concentrations. Scale bar: 50 µm. (B) Surface SEM image (viewed at a 45° tilt) and cross-sectional SEM image for 12 wt% SEBS concentration. The left scale bar is 50 µm, and the right scale bar is 2 µm.

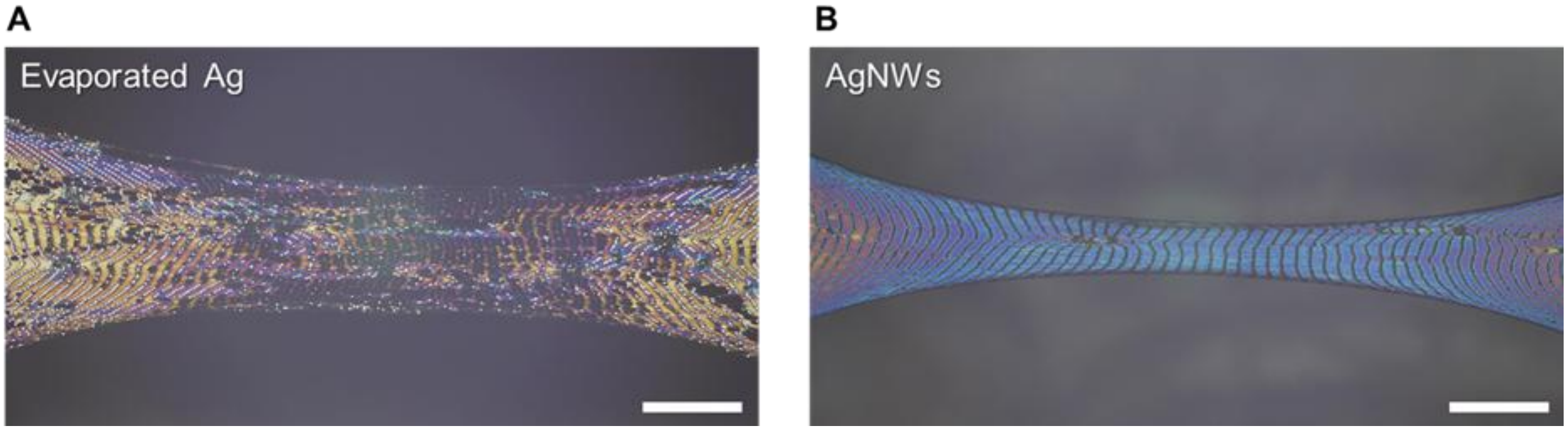

Fig. S8. Photographs of the Deposited Silver Sample and the AgNWs Sample at 500% Strain. (A) Image of the deposited silver sample during stretching. (B) Image of the AgNWs sample during stretching. Scale bar: 2 mm.

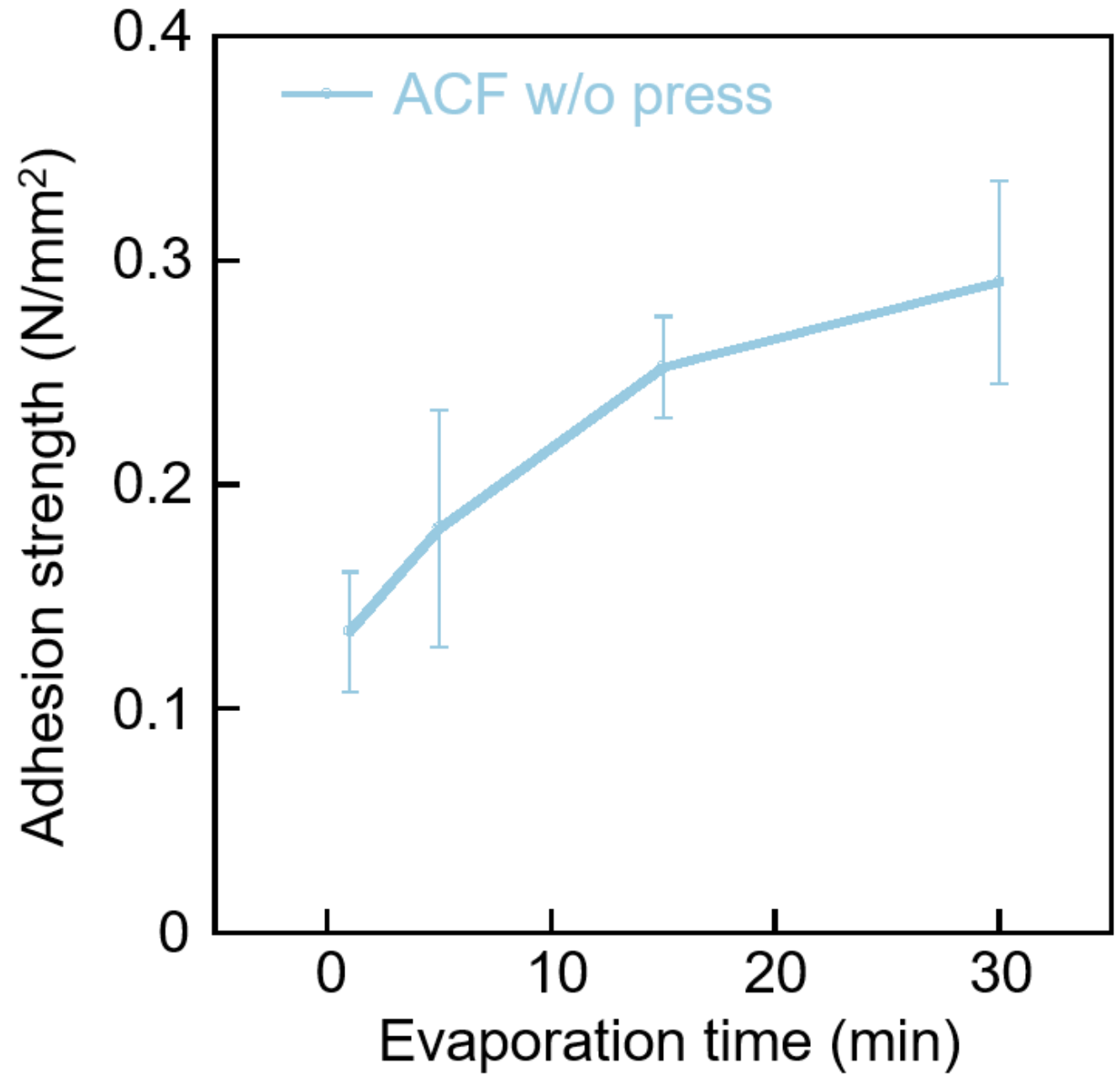

Fig. S9. Time-Dependence of Adhesion Strength in the Wet-Volatile Process. Time-dependent change in bonding force established by ethanol volatilization in the wet-volatile process, evaluated using the shear strength test during bonding of 12.5-µm-thick polyimide films with the ultrathin stretchable ACF. The mean and standard deviation of three samples are shown.

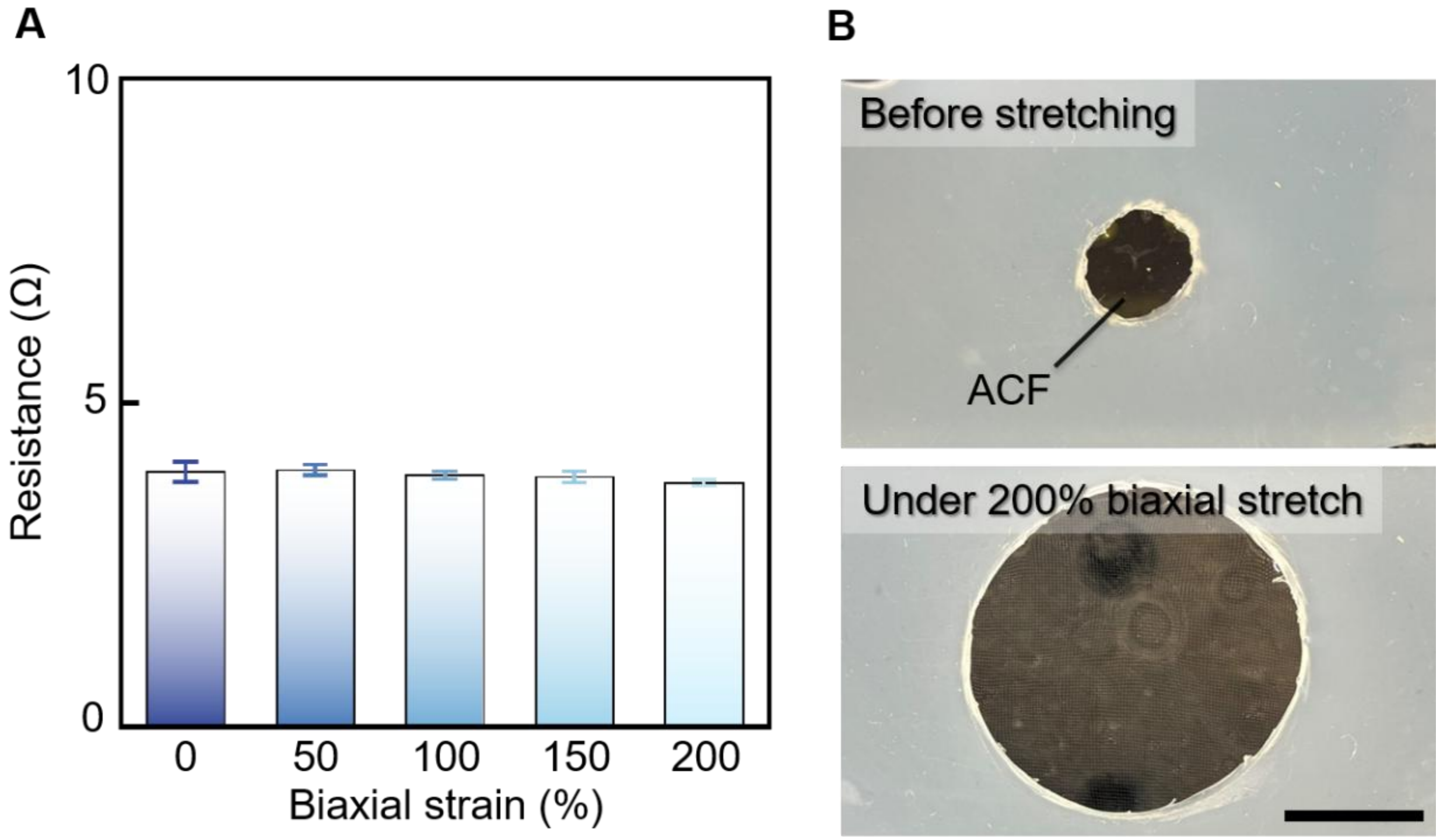

Fig. S10. Electrical Resistance and Deformation of the Ultrathin Stretchable ACF under Biaxial Stretching. (A) Through-thickness electrical resistance of the ultrathin stretchable ACF was measured while being bi-axial stretched and sandwiched between conductive wiring films. Bars represent the mean of three samples, and the standard deviation is shown by lines. (B) Optical images of the ultrathin stretchable ACF before stretching (0%) and after 200% bi-axial stretching. Scale bar: 10 mm.

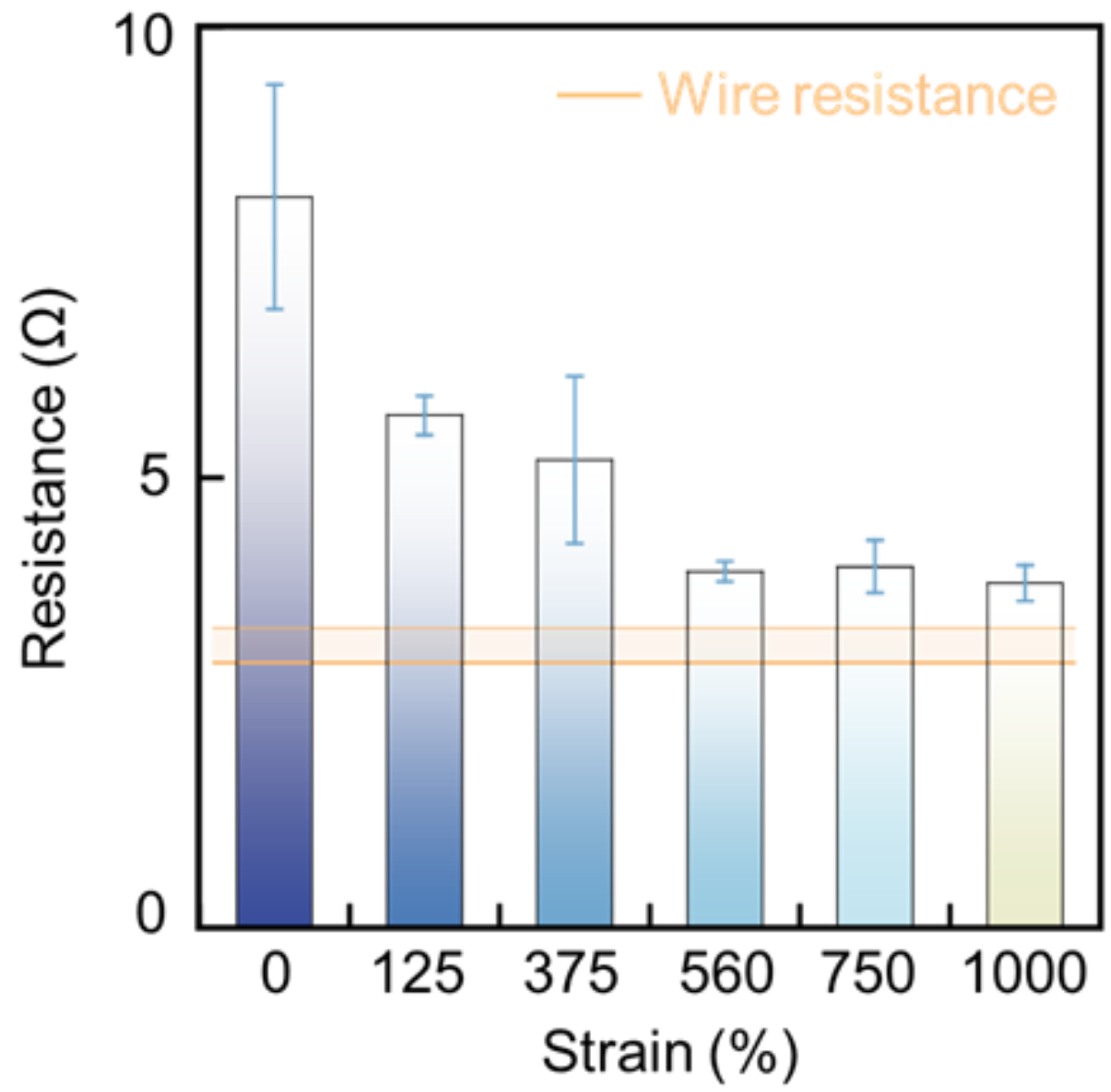

Fig. S11. Electrical Resistance of 12 wt% SEBS ACF During Stretching. The resistance of ACF fabricated with 12 wt% SEBS was measured while being stretched and sandwiched between conductive wiring films. Bars represent the mean of three samples, and the standard deviation is shown by lines. The upper and lower bounds of the standard deviation for the wiring resistance of the three samples are indicated by yellow lines.

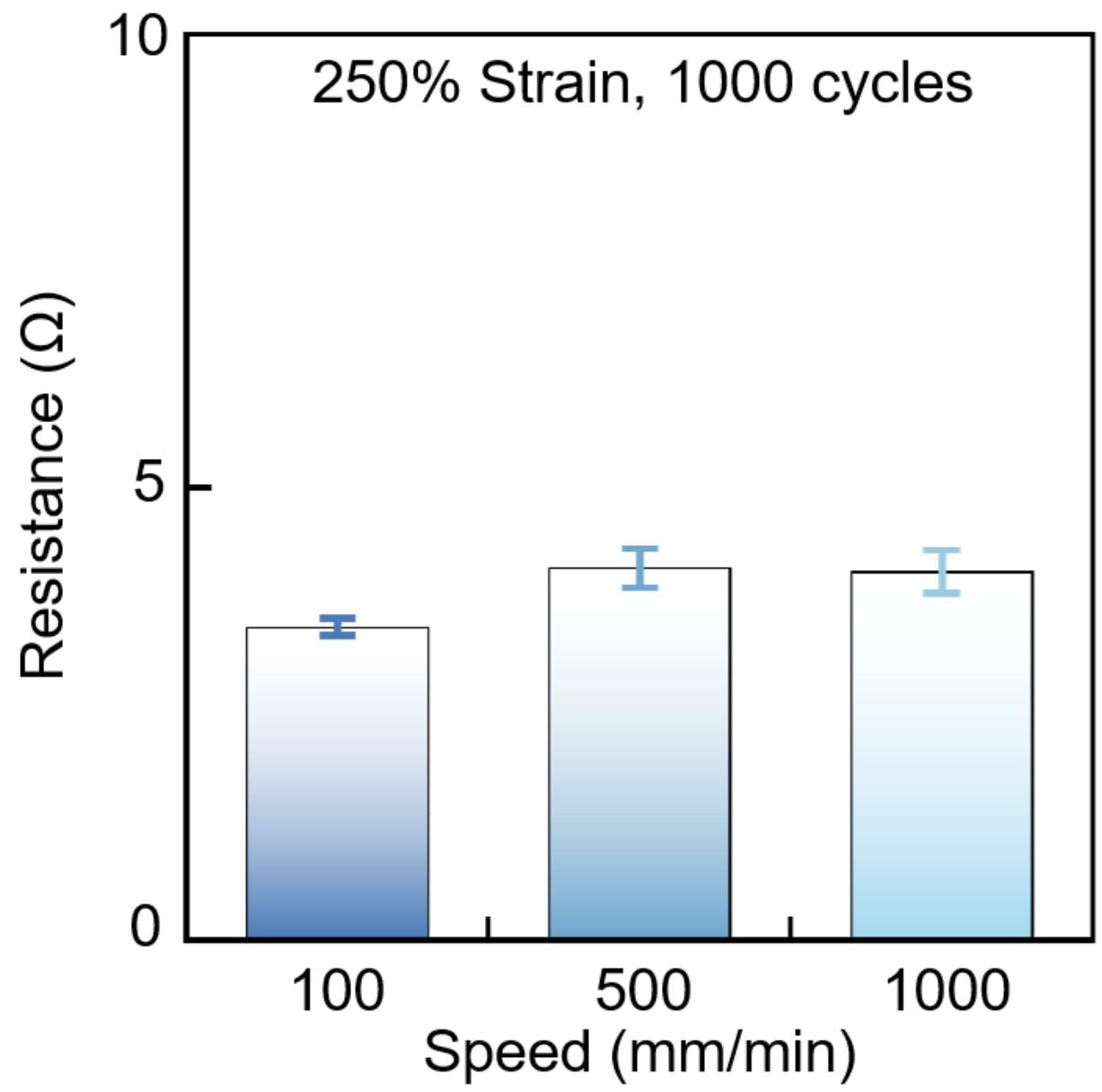

Fig. S12. Electrical Resistance of the Ultrathin Stretchable ACF After High-Speed Cycling. Electrical resistance of parylene-based Cr/Au films bonded with an ultrathin stretchable ACF area of 2 mm × 2 mm in a relaxed state after 1,000 stretching cycles performed at speeds ranging from 100 to 1000 mm/min. Bars represent the mean peak values (n = 3), and the standard deviation is shown by lines.

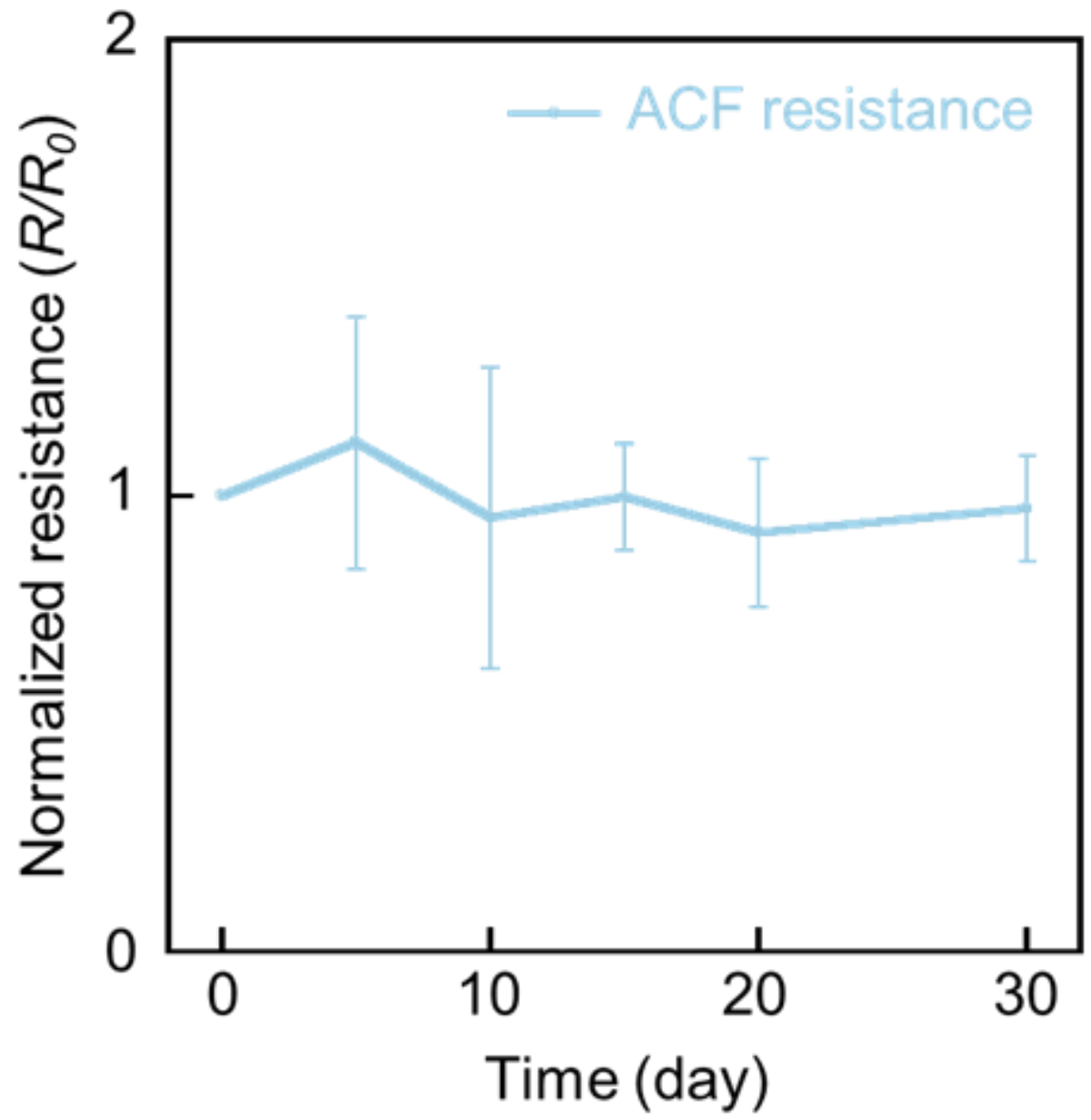

Fig. S13. Electrical Stability in Air After Bonding Conductive Wiring Films with ACF. Change in electrical resistance from the initial value for a sample where conductive wiring films were bonded with ACF over a 5 mm by 5 mm area, after 30 days of storage in air. The mean and standard deviation of three samples are shown.

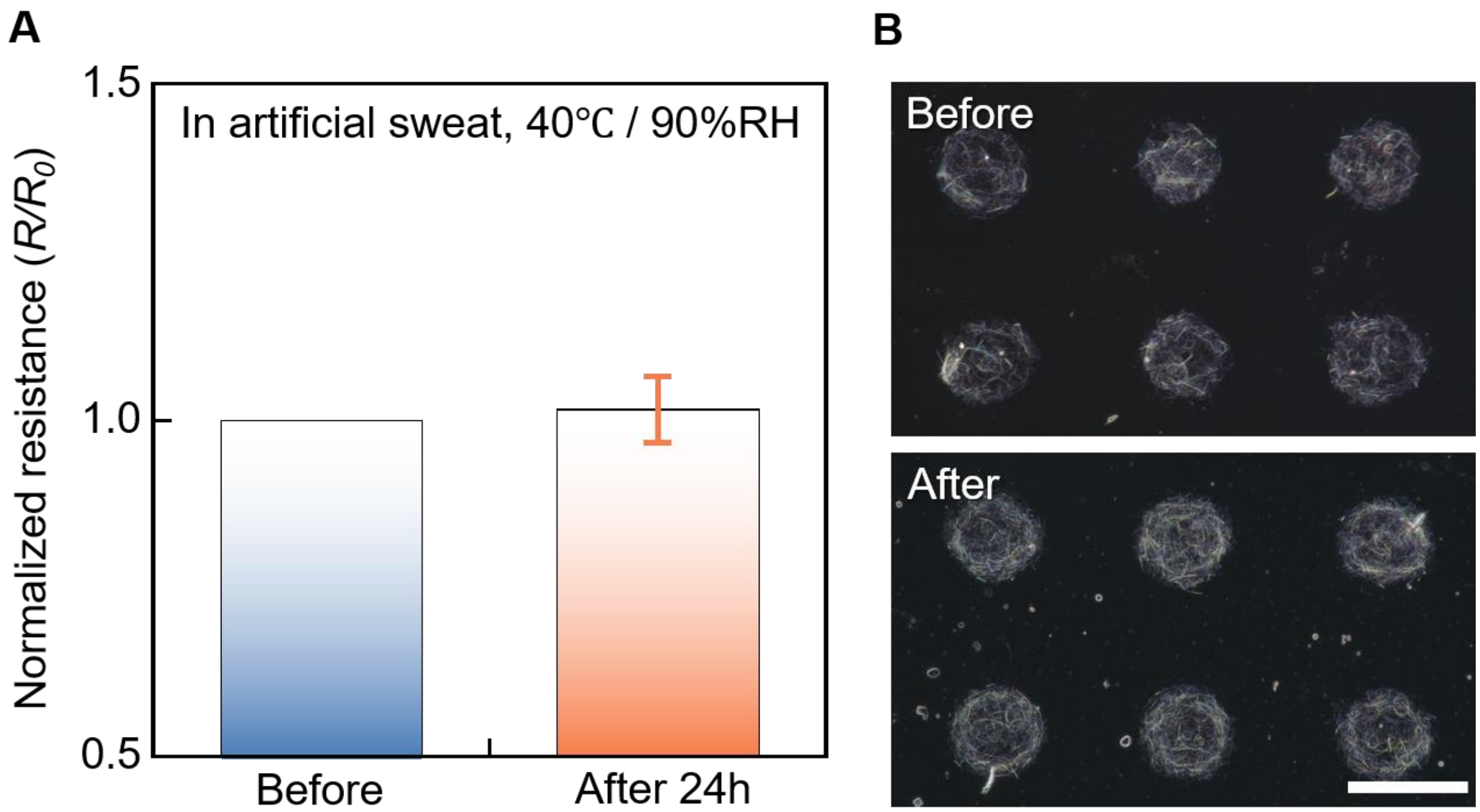

Fig. S14. Stability of the Ultrathin Stretchable ACF in Contact with Artificial Sweat. (A) Electrical resistance was measured after 24 hours of exposure to artificial sweat at 40°C / 90%RH. Bars represent the mean resistance of six samples (n = 6), normalized to the resistance measured before the test, and the standard deviation is shown by lines. (B) Optical images of the ultrathin stretchable ACF before (top) and after 24 hours of exposure to artificial sweat (bottom). Scale bar: 50 µm.

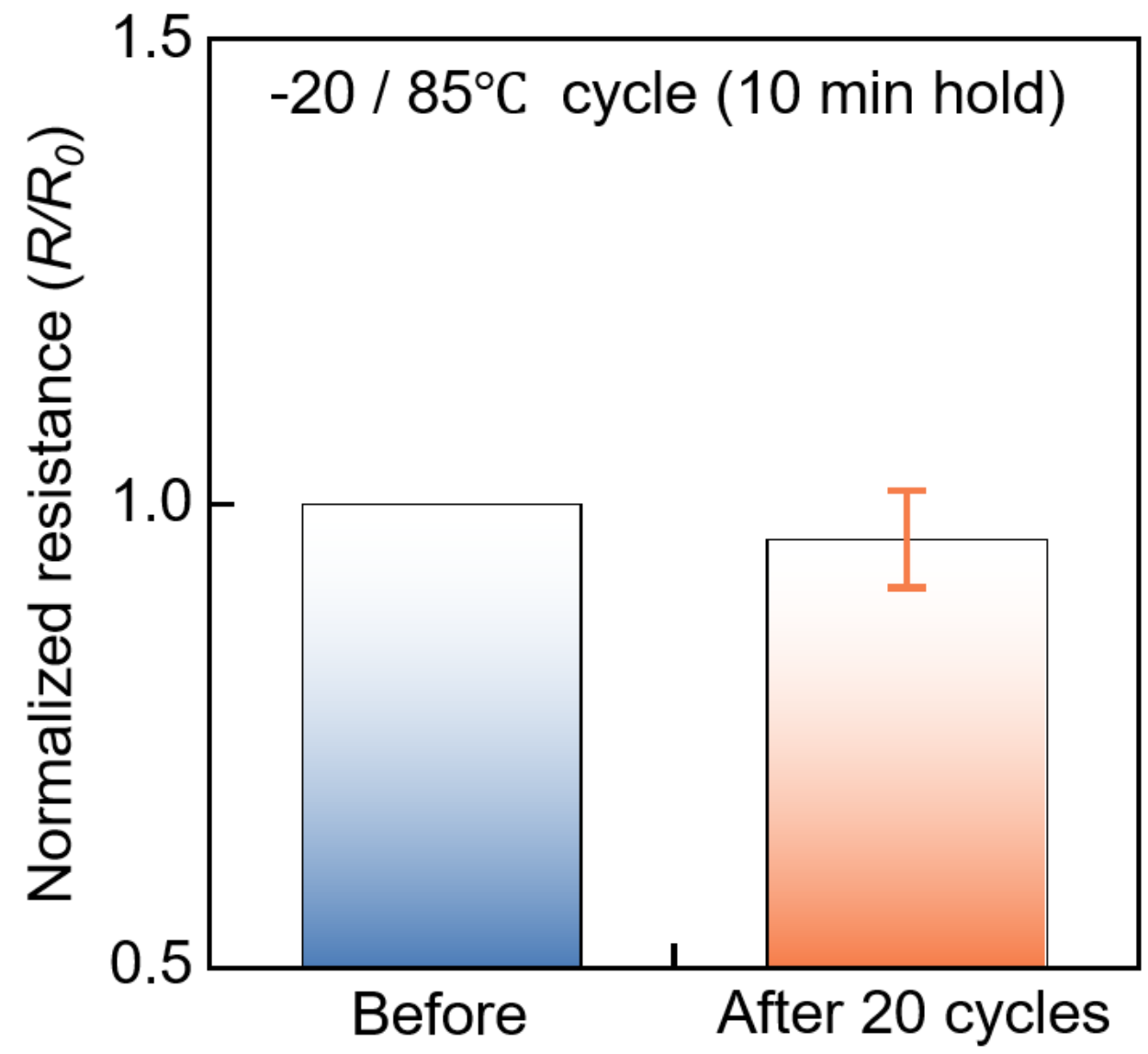

Fig. S15. Electrical Stability of the Ultrathin Stretchable ACF After Temperature Cycles. (A) Electrical resistance was measured after a temperature cycling test (–20°C/+85°C, 10 min hold at each temperature, 20 cycles). Bars represent the mean resistance of six samples (n = 6), normalized to the resistance measured before the test, and the standard deviation is shown by lines.

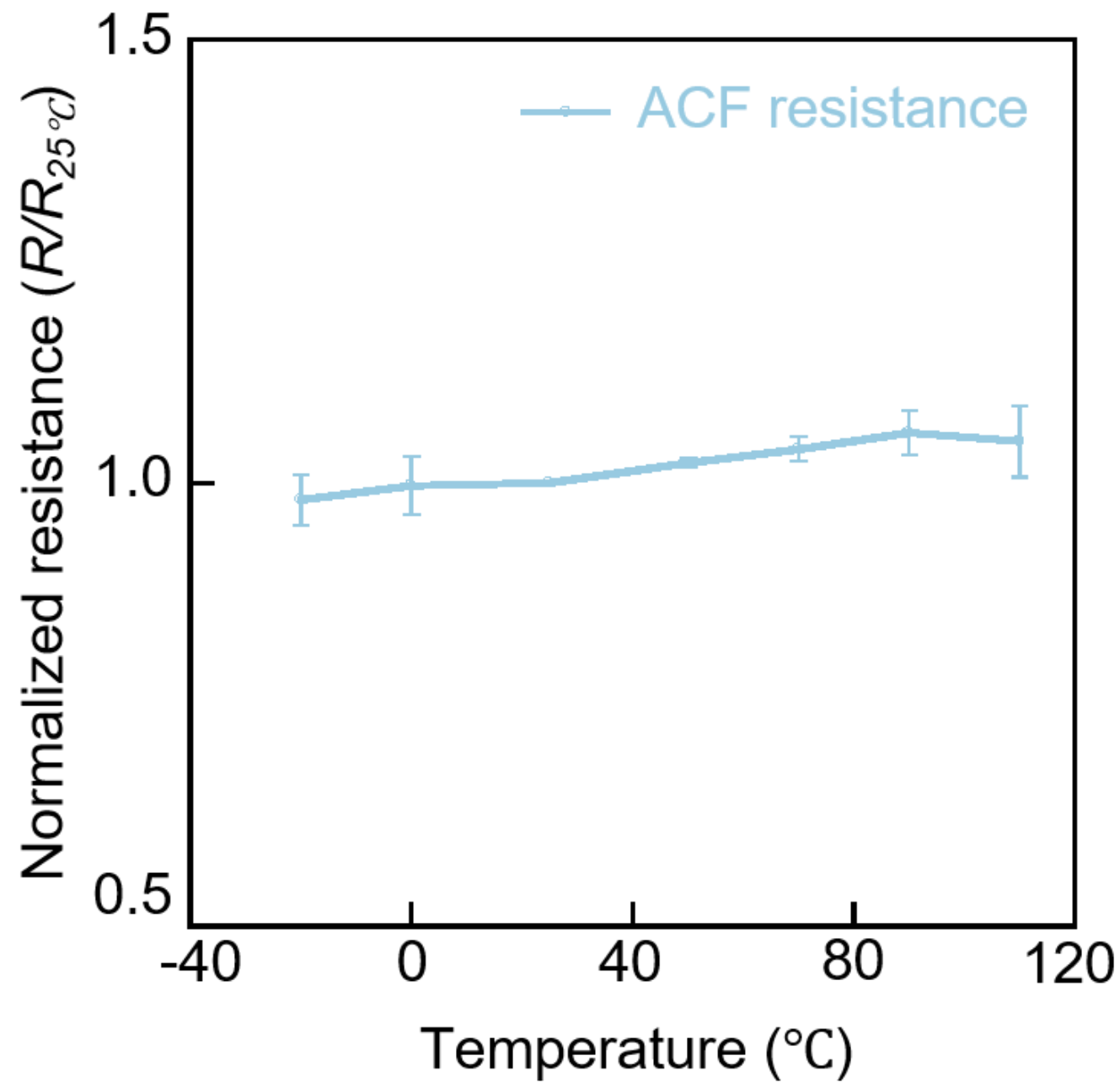

Fig. S16. Electrical Stability of the Ultrathin Stretchable ACF Under Wide Temperature Range. Electrical resistance of the Ultrathin Stretchable ACF was measured across a temperature range from –20°C to +110°C, normalized to the resistance measured at 25°C. The mean and standard deviation of three samples are shown.

Table S1. Water Vapor Transmission Rate (WVTR) of Various Films. The mean and standard deviation were calculated from three samples.

	Open	SEBS-3wt%	ACF-3wt%	SEBS-12wt%	ACF-12wt%	Control
Water vapor transmission rate ($g/m^2/day$)	4746±23.09	1346±299.2	1227±251.4	180.8±26.48	278.0±175.3	183.7±6.66

Table S2. Comparison with Previous Studies. Film thickness is estimated from figures in the literature if not explicitly stated in the text. For WVTR, bonding pressure and bonding strength unspecified values are left blank.

	Thickness (μm)	Conductive stretchability (%)	WVTR ($g/m^2/day$)	Bonding temperature (°C)	Bonding pressure (MPa)	Bonding time	Bonding mechanism	Bonding strength	Materials
This	0.3	500	1200	25	0	1-30min	Wet-volatile process	Shear: 0.28 MPa, T-peel: 9.4×10^{-3} N/mm [Shear: 1.1 N / (2 mm × 2 mm), T-peel: 1.9×10^{-2} N / 2 mm]	AgNWs, SEBS
(29)	3	0	-	180	0.05-0.6	10 sec	hot-press	T-peel: > 0.65 N/mm [> 9.8 N /15 mm]	Au, Epoxy
(30)	150	199.12	-	150	-	1 hour	hot-press	Shear: 25.22MPa [(Force) / (10 mm × 5 mm)]	Mxene, Epoxy
(32)	1.2	60	-	110	-	90 sec	hot-press	-	EGaIn, Epoxy
(33)	10	180	-	25	0	1 min	UV curing	90° peel: 0.254 N/mm [(Force) / 20 mm]	Au, SEBS/ FP-AZ4 etc.
(35)	0.5	0	-	200	0.1	>1 hour	Direct spin-coating UV curing heating in Ar	3.0×10^{-3} N/mm	In, Epoxy Acrylate

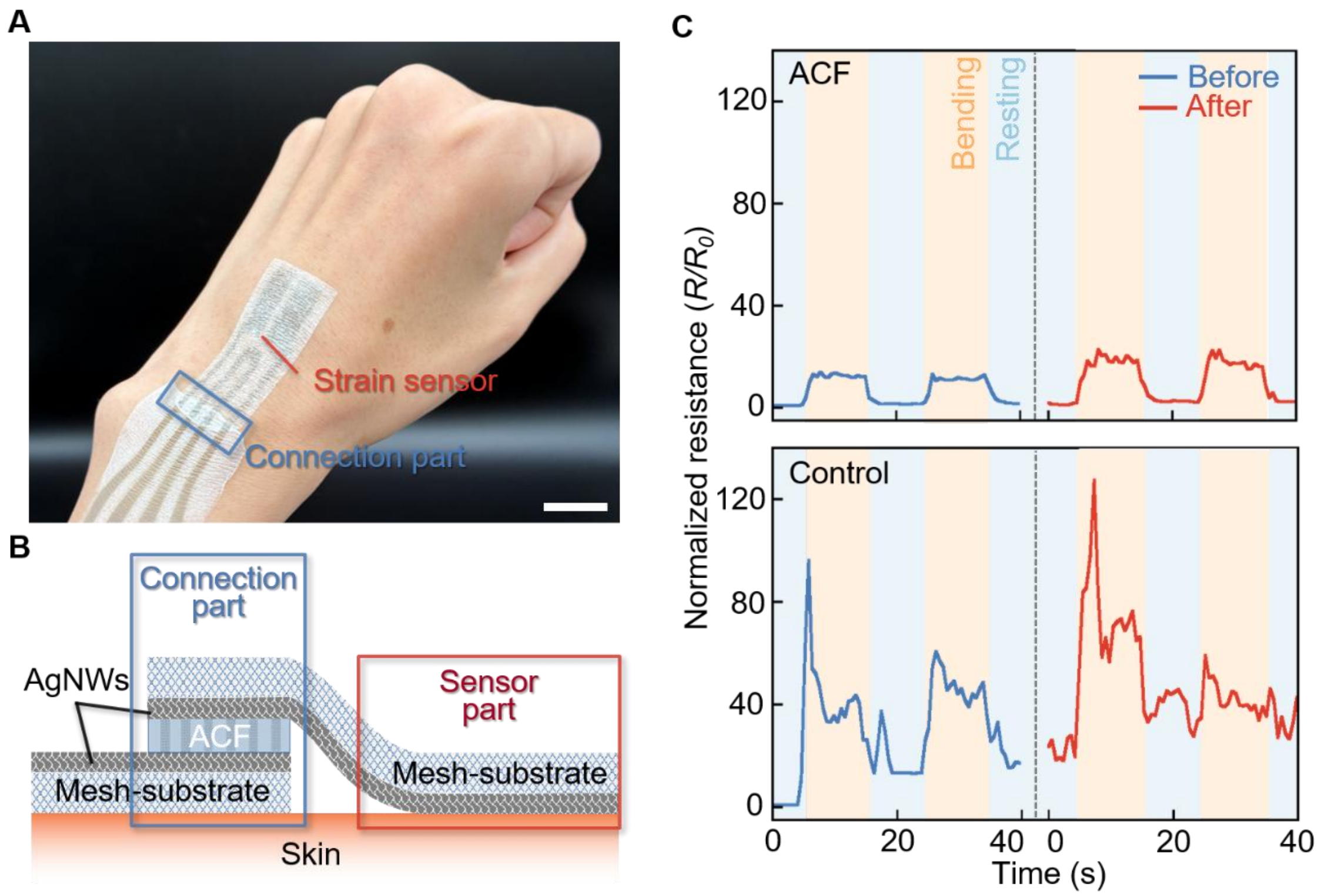

Fig. S17. Wrist bending measurement with the ultrathin stretchable ACF-interconnected mesh sensor. (A) Image of the mesh-substrate strain sensor integrated onto the wrist. Scale bar: 20 mm. (B) Schematic illustration of the device structure. (C) Normalized resistance plots of the device bonded with the ultrathin stretchable ACF (top) and the control device with the junction stretchability restricted (bottom).

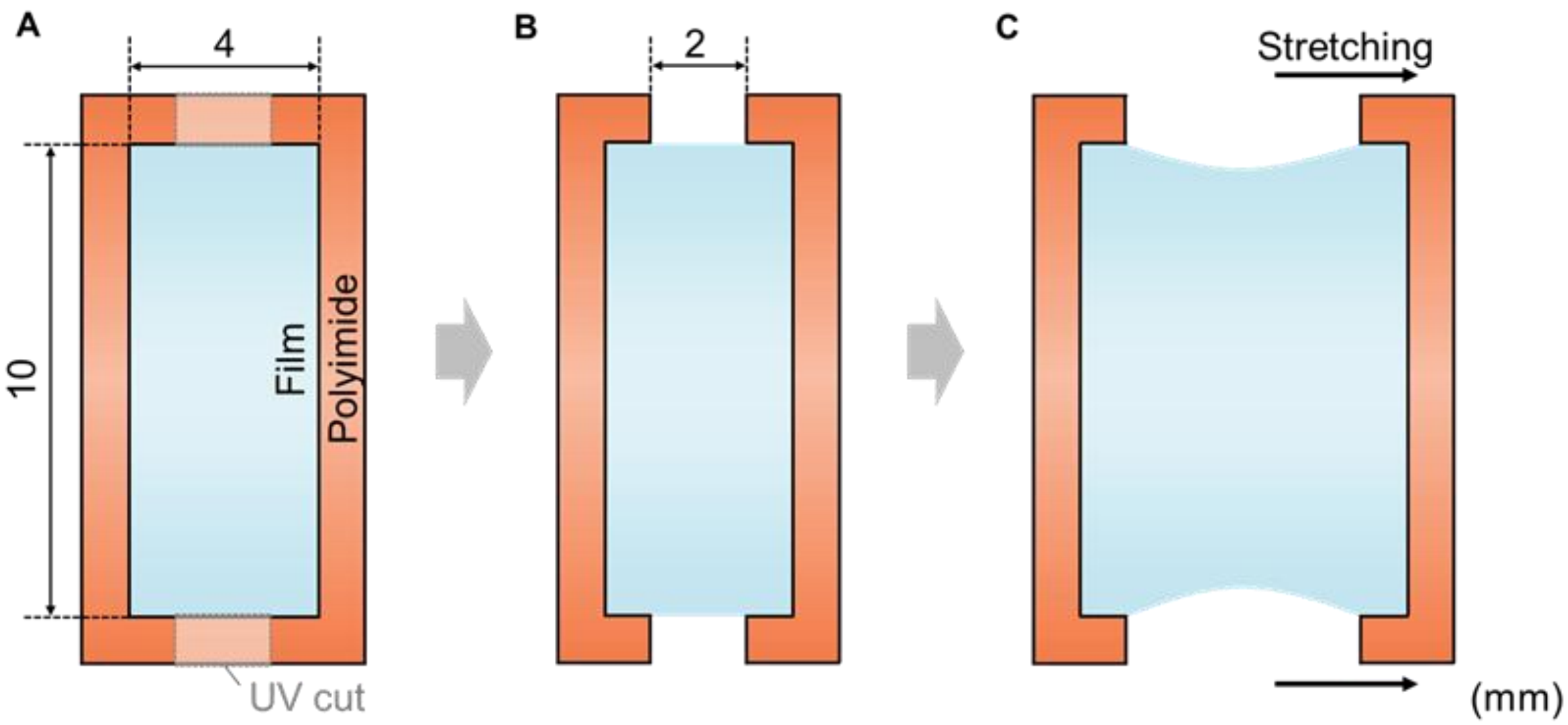

Fig. S18. Schematic Diagram Illustrating the Method for the Film Stretching Experiment. (A) A polyimide film jig is attached to a 4 mm by 10 mm film, and the center of the jig on the short side is cut by a UV laser. (B) The 2 mm film becomes freestanding. The center-to-center distance of 4 mm at this point is used as the gauge length for calculating the strain. (C) Tension is applied to the film by pulling the film together with the jig. All dimensions are in mm.

Movie S1. Elongation Behavior of ACF.

Elongation of the ultrathin stretchable ACF under manual stretching.

Movie S2. Tensile Fracture of the Bonded Films.

A sample, where two 4-µm-thick parylene films were bonded with ultrathin stretchable ACF over a 2 mm by 2 mm area, subjected to tension.

Movie S3. LED Light Emission Demo Under Deformation.

A sample where two LEDs were integrated with EGaIn conductive wiring using ultrathin stretchable ACF was stretched up to 500% strain and then returned to its original length.

Movie S4. On-Skin ACF Bonding Demonstration.

Demonstration of on-skin interconnection between stretchable devices using the ultrathin stretchable ACF, showcasing stable operation during wrist movement.
